# Cell-surface major vault protein promotes cancer progression through harboring mesenchymal and intermediate circulating tumor cells in hepatocellular carcinomas

**DOI:** 10.1038/s41598-017-13501-1

**Published:** 2017-10-16

**Authors:** Hyun Min Lee, Jae Won Joh, Se-Ri Seo, Won-Tae Kim, Min Kyu Kim, Hong Seo Choi, So Young Kim, Young-Joo Jang, Dong Hyun Sinn, Gyu Seong Choi, Jong Man Kim, Choon Hyuck David Kwon, Hee Jin Chang, Dae Shick Kim, Chun Jeih Ryu

**Affiliations:** 1 Department of Integrative Bioscience and Biotechnology, Institute of Anticancer Medicine Development, Sejong University, Seoul, Korea; 2 Department of Surgery, Samsung Medical Center, Sungkyunkwan University School of Medicine, Seoul, Korea; 3 Department of Nanobiomedical Science, BK21 PLUS Global Research Center for Regenerative Medicine, Dankook University, Cheonan, Korea; 4 Department of Medicine, Samsung Medical Center, Sungkyunkwan University School of Medicine, Seoul, Korea; 5 Center for Colorectal Cancer, Research Institute and Hospital of National Cancer Center, Goyang-si, Korea; 6 Department of Pathology, Samsung Medical Center, Sungkyunkwan University School of Medicine, Seoul, Korea

## Abstract

Circulating tumor cells (CTCs) play a major role in the metastasis and recurrence of hepatocellular carcinoma (HCC). Here, we found that major vault protein (MVP) is expressed on the surface of HCC cells and further induced under stressful environments. MVP knockdown reduces cell proliferation and induces apoptosis in HCC cells. Treatment of HCC cells with anti-MVP antibody (α-MVP) recognizing cell-surface MVP (csMVP) inhibits cell proliferation, migration, and invasion. csMVP-positive HCC cells have a higher clonogenic survival than csMVP-negative HCC cells, and treatment of HCC cells with α-MVP inhibits clonogenic survival, suggesting that csMVP contributes to HCC cell survival, migration, and invasion. The function of csMVP is mediated through mTOR, FAK, ERK and Akt signaling pathways. csMVP-positive CTCs are detected in HCC patients (89.7%) but not in healthy donors, and the number of csMVP-positive CTCs is further increased in patients with metastatic cancers. csMVP is exclusively detectable in CTCs with mesenchymal phenotype or intermediate phenotype with neither epithelial nor mesenchymal markers, suggesting that csMVP-associated survival and metastatic potential harbor CTCs with nonepithelial phenotypes. The results suggest that csMVP promotes cancer progression and serves as a surface marker for mesenchymal and intermediate CTCs in patients with HCC and metastatic cancers.

## Introduction

Hepatocellular carcinoma (HCC) is the third most common cause of cancer mortality^1^. Long-term survival is achieved when the HCCs are removed by hepatic resection, transarterial chemoembolization, and liver transplantation^2^. However, high recurrence rate (>70%) is generally observed in HCC patients due to intrahepatic and extrahepatic metastases even after curative treatments^3^, suggesting that HCC cells have a special ability to survive, remain dormant, and initiate recurrence. Circulating tumor cells (CTCs) are considered to be the initiators of metastasis and recurrence^4^. Therefore, CTC analysis in patients with HCC may provide meaningful information on how tumor cells survive, remain dormant, and initiate recurrence during cancer metastasis and recurrence.

CTCs should remain alive in the bloodstream and other tissues until establishing metastasis or recurrence after shedding into the bloodstream by the primary tumor. They should also escape anoikis and evade host immune defenses. Although it is not clear how CTCs survive under such hostile environments, epithelial-mesenchymal transition (EMT) is regarded as one mechanism that enables CTCs to avoid apoptosis, anoikis, and immune surveillance^5^. Epithelial cancer cells are able to convert into invasive and motile mesenchymal cancer cells through the EMT process during metastasis^6,7^. However, the only approved CTC detection system is to use epithelial cell adhesion molecule (EpCAM) that is downregulated on invasive CTCs during the EMT process^7^. EMT-phenotypic CTCs are abundantly detected in patients with HCC, although HCC cells originate from epithelial cells^8^. Therefore, the low number of CTCs is detected in HCC patients by the current CTC detection system^9–11^. To detect and capture CTCs that are missed under the current CTC detection system, therefore, it is necessary to discover novel surface markers on CTCs with nonepithelial phenotype in HCC patients.

Vaults are multi-subunit ribonucleoprotein particles that are involved in nuclear-cytoplasmic transport. Major vault protein (MVP) is the main component of vaults and forms the outer shell of vaults^12^. Although MVP is distributed in diverse normal tissues, its expression is upregulated in multidrug-resistant cancer cells^12^. Enhanced expression of MVP has been associated with chemotherapy failure and radiation resistance during cancer progression^13^. MVP is a HCC diagnostic biomarker that can distinguish HCC tissues from normal liver^14^. The tumor promoting potential of MVP is based on MVP-mediated stabilization of the epidermal growth factor receptor (EGFR)/phosphatidyl-inositol-3-kinase (PI3K)-mediated migration and survival pathways in human glioblastoma multiform cells^15^. However, how MVP contributes to the derivation of CTCs and cancer metastasis remains elusive. Here, we found for the first time that MVP is expressed on the cell surface of HCC cells but not on the surface of hepatocytes. Cell surface MVP (csMVP) was induced under stressful environments, such as serum starvation, DNA damage, and detachment stress. To understand the role of csMVP on HCC cells, we treated HCC cells with polyclonal anti-MVP antibodies (α-MVP) recognizing csMVP. Treatment with α-MVP inhibited HCC cell proliferation, migration, and invasion. Cell sorting further revealed that csMVP-positive HCC cells have a higher clonogenic survival than csMVP-negative HCC cells. Treatment with α-MVP inhibited the clonogenic survival of HCC cells, suggesting that csMVP contributes to the clonogenic survival of HCC cells. The function of csMVP was mediated through mTOR, FAK, ERK and Akt signaling pathways, which are associated with cancer cell survival and metastasis. Therefore, the role of csMVP was further investigated by analyzing CTCs in patients with HCC and metastatic cancers. The findings are the first report showing that csMVP is a novel surface marker for mesenchymal and intermediate CTCs in patients with HCC and metastatic cancers. We also discuss and propose the biological significance of csMVP-positive CTCs in HCC and metastatic cancers.

## Results

### csMVP is expressed on HCC cells and induced under stressful environments

**Figure 1:**
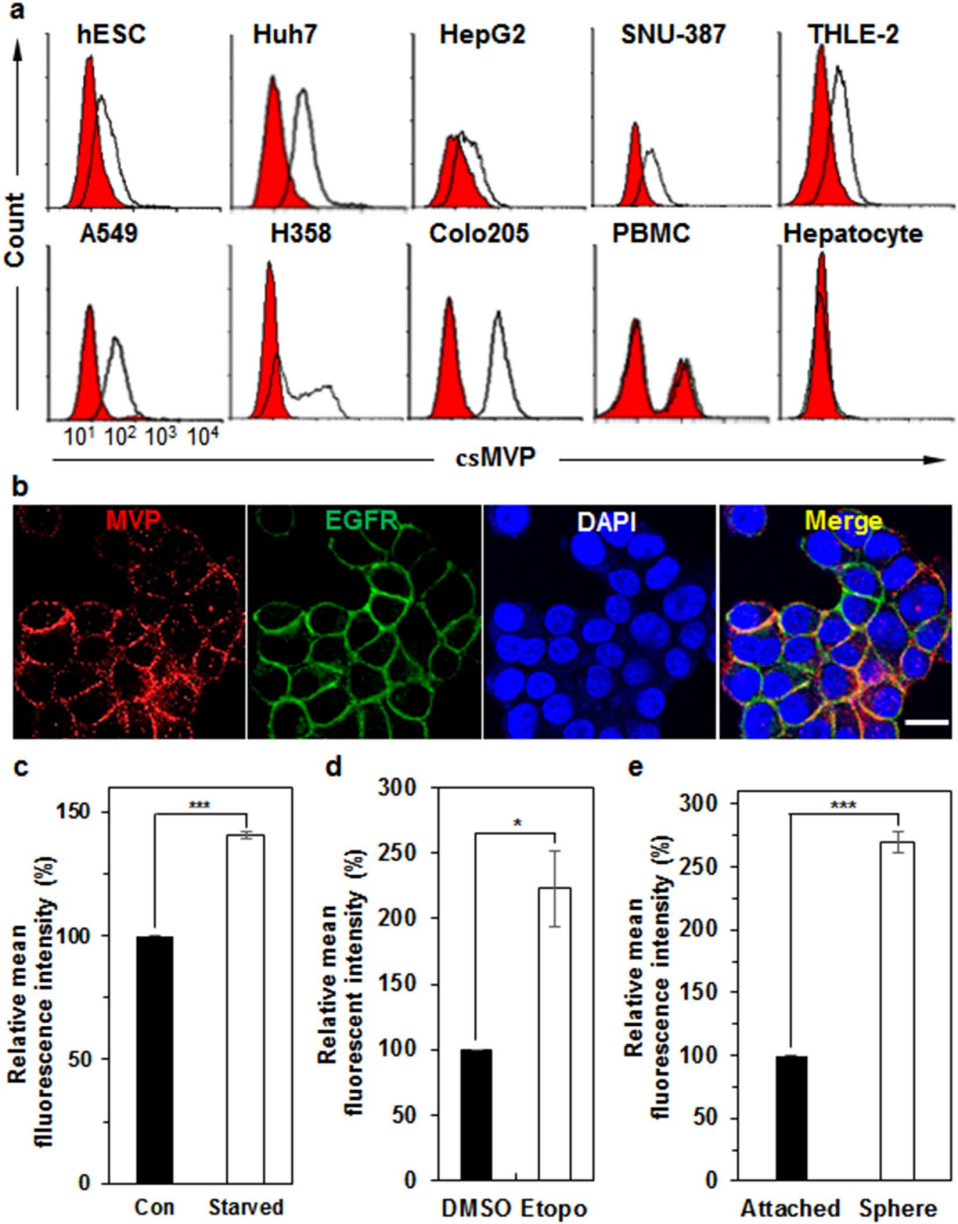
csMVP is expressed on HCC cells but not on normal hepatocytes, and is induced under stressful environments, such as serum starvation, DNA damage, and detachment stress. (**a**) Flow cytometry analysis of HCC cells (Huh7, HepG2, and SNU-387), immortalized hepatocytes (THLE-2), NSCLC cells (A549 and H358), colorectal cancer cells (Colo205), PBMCs, and primary hepatocyte with α-MVP. (**b**) Immunocytochemical analysis of Huh7 cells with α-MVP and EGFR antibodies. Huh7 cells were incubated with α-MVP (red) and anti-EGFR (green) antibodies. Nuclei were labelled with DAPI. The scale bar is 20 μm. **(c**) Induced expression of csMVP on the surface of serum-starved Huh7 cells. Relative expression of csMVP was measured by mean fluorescence intensities (MFIs) of flow cytometric analysis of normal (con) or serum-starved Huh7 cells (starved). ***p < 0.005. (**d**) Induced expression of csMVP on the surface of etoposide-treated Huh7 cells. Relative expression of csMVP was measured by MFIs of flow cytometric analysis of DMSO or Etopo-treated Huh7 cells. *p < 0.05. (**e**) Induced expression of csMVP on Huh7 sphere cells. Relative expression of csMVP was measured by MFIs of flow cytometric analysis of attached or sphere cultures of Huh7 cells. ***p < 0.005.

Although MVP is expressed in the cytoplasm and nucleus, and is ubiquitously expressed in most tissues^16,17^, MVP is a HCC diagnostic biomarker that can be used to distinguish HCC tissues from normal liver^14^. While searching for novel oncofetal antigens on human embryonic stem cells (hESCs), we accidentally found that MVP is detected on the cell surface of hESCs with rabbit polyclonal antibodies against the C-terminal region of MVP (α-MVP) (Fig. 1a). Cell surface-expressed MVP (csMVP) was also detected on the surface of various HCC cell lines (Huh7, HepG2, and SNU-387), transformed hepatocytes (THLE-2), and some cancer cell lines (A549, H358, and Colo205) with α-MVP (Fig. 1a). However, csMVP was not detected in primary hepatocytes and PBMCs (Fig. 1a). Multi-color immunocytochemistry also showed that both csMVP and EGFR are colocalized at the cell surface (Fig. 1b). To further prove whether MVP is really expressed on the cell surface, Huh7 cell were stained with five commercially available anti-MVP antibodies. Antibodies recognizing the C-terminal region of MVP were able to recognize MVP on the cell surface (Supplementary Fig. 1a). To figure out how MVP translocates to the cell surface, we treated Huh7 cells with some chemical inhibitors of cancer cell signaling pathways. While LY294002, a Akt inhibitor, did not decrease cell surface expression of MVP, U0126 (ERK inhibitor) and rapamycin (mTOR inhibitor) significantly decreased cell surface expression of MVP (Supplementary Fig. 1b). Taken together, the results suggest that MVP is expressed on the surface of HCC cells and cell surface translocation of MVP is regulated by ERK and mTOR signaling.

MVP induction represents a general stress-induced protection signal in neuroectodermal cells^18^. To look at whether csMVP is associated with HCC cell survival under stressful conditions, the expression of csMVP was examined when Huh7 cells were cultured under serum starvation. The expression of csMVP was increased by approximately 41% (Fig. 1c and Supplementary Fig. 2a). The expression of csMVP was also increased by approximately 123% with etoposide, an inhibitor of topoisomerase II (Fig. 1d and Supplementary Fig. 2b). The expression of csMVP was also increased by approximately 170% in the sphere culture of Huh7 cells (Fig. 1e and Supplementary Fig. 2c). Similar results were also obtained with A549 cells (Supplementary Fig. 2d–f). Thus, cell surface expression of MVP is induced under stressful environments.

### csMVP contributes to HCC cell proliferation, migration, and invasion

**Figure 2:**
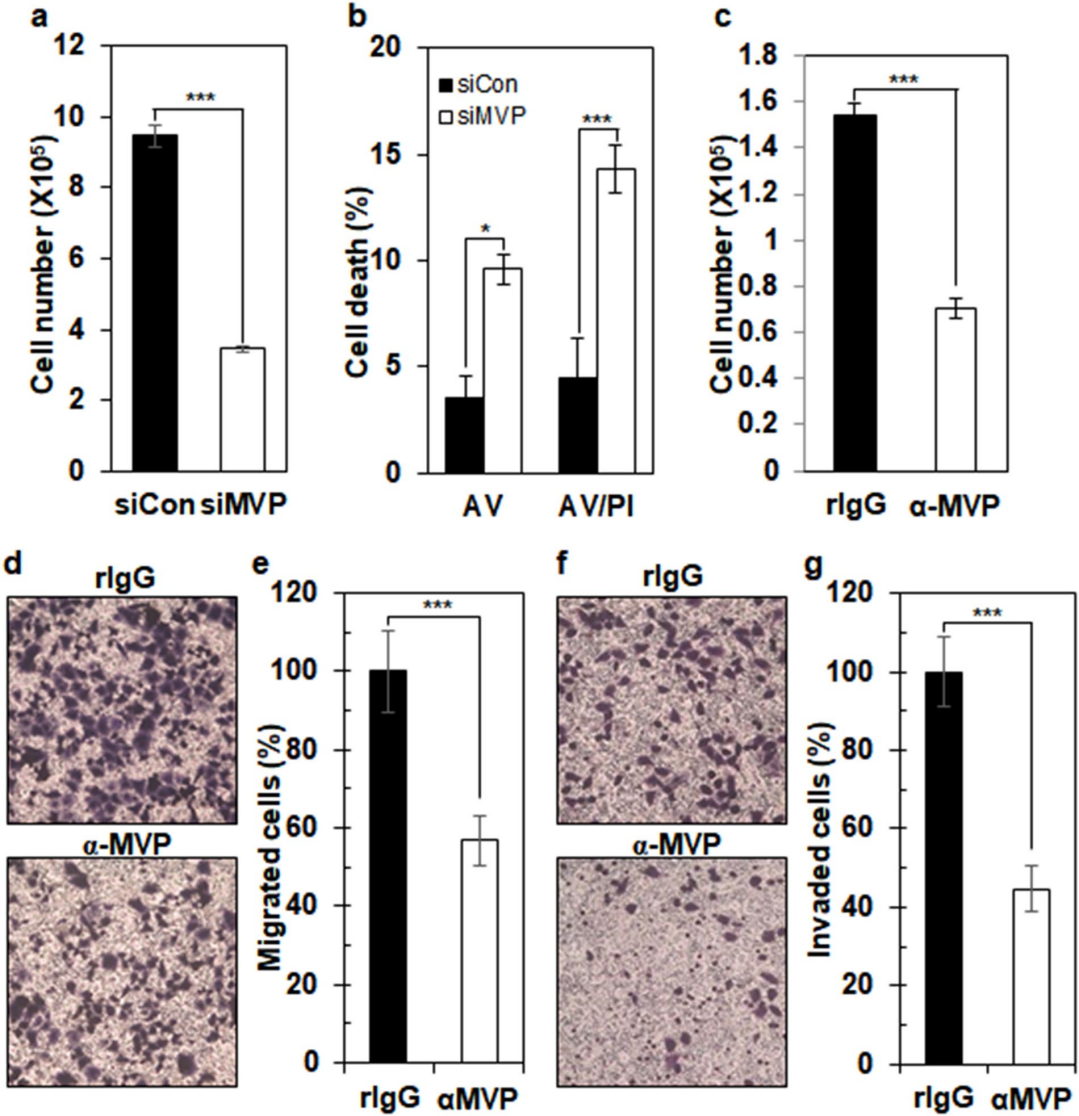
Cell surface expression of MVP contributes to HCC cell proliferation, migration, and invasion. (**a**) Measurement of cell proliferation of control or MVP knockdown Huh7. The cell numbers of control or MVP knockdown Huh7 cells were determined (*n* = 4) by trypan blue exclusion assay. ***p < 0.005. (**b**) Apoptosis analysis of MVP knockdown Huh7 cells. Control or MVP siRNA-treated Huh7 cells were stained (*n* = 5) with PI and annexin V-FITC. Shown are percentages of single annexin V-positive (AV) and both annexin V- and PI-positive cells (AV/PI). *p < 0.05; ***p < 0.005. (**c**) Measurement of cell proliferation of α-MVP-treated Huh7 cells. Huh7 cells were incubated with control rIgG or α-MVP for 48 hours, and the cell numbers were determined (*n* = 3) by trypan blue exclusion assay. ***p < 0.005. (**d**) Effect of α-MVP treatment on HCC cell migration. In transwell-migration assays, Huh7 cells were incubated with rIgG or α-MVP on the top of the transwell chambers. The degree of transwell filter migration was measured by staining cells at the bottom of filters with crystal violet. (**e**) Graphic presentation of d. Stained cells were counted by image analysis software. Shown is relative percentage of α-MVP-treated Huh7 cells (*n* = 12; ***p < 0.005). (**f**) Effect of α-MVP treatment on HCC cell invasion. The upper and lower sides of the chamber filters were coated with Matrigel, and Huh7 cells were incubated with rIgG or α-MVP on top of the coated transwell chambers. The degree of transwell filter invasion was measured by staining cells at the bottom of filters with crystal violet. (**g**) Graphic presentation of f. Stained cells were counted by image analysis software. Shown is relative percentage of α-MVP-treated Huh7 cells (*n* = 12; ***p < 0.005).

To study the function of MVP in HCC cells, we first depleted MVP in Huh7 cells by siRNA technology. MVP protein expression was decreased by approximately 95% in MVP knockdown Huh7 cells (Supplementary Fig. 3a,b). Cell surface expression of MVP was also decreased by approximately 44% in MVP knockdown Huh7 cells (Supplementary Fig. 3c,d). In this context, MVP knockdown decreased cell proliferation in Huh7 cells by approximately 63% (Fig. 2a). Annexin V-positive cells were increased by approximately 6% in MVP knockdown Huh7 cells, and both annexin V- and propidium iodide (PI)-positive cells were increased by approximately 10% (Fig. 2b and Supplementary Fig. 3e). Thus, MVP depletion inhibits HCC cell proliferation by inducing apoptosis.

In order to dissect the role of csMVP in Huh7 cells, Huh7 cells were treated with α-MVP. Treatment with α-MVP decreased Huh7 cell proliferation by approximately 54% (Fig. 2c), although treatment with α-MVP did not induce apoptosis (Supplementary Fig. 4a,b). Treatment with another α-MVP recognizing csMVP also decreased Huh7 proliferation by approximately 63% (Supplementary Fig. 4c). Treatment with α-MVP also decreased cell proliferation in another HCC cell line (HepG2) by approximately 54% (Supplementary Fig. 4d). Taken together, the results suggest that csMVP contributes to HCC cell proliferation.

csMVP was also induced during the sphere culture of Huh7 cells (Fig. 1e). Suspension sphere cultures of cancer cells display EMT signatures and invasive and metastatic potential of cancer cells^19,20^. To look at whether csMVP is associated with the migratory potential of HCC cells, Huh7 cells treated with α-MVP were subjected to transwell-migration assays. Treatment with α-MVP decreased the migratory potential of Huh7 cells by approximately 43% (Fig. 2d,e). Treatment with α-MVP also decreased the invasive potential of Huh7 cells by approximately 55% (Fig. 2f,g). Thus, csMVP positively regulates the migratory and invasive potential of HCC cells.

### csMVP supports the clonogenic survival of HCC cells

**Figure 3:**
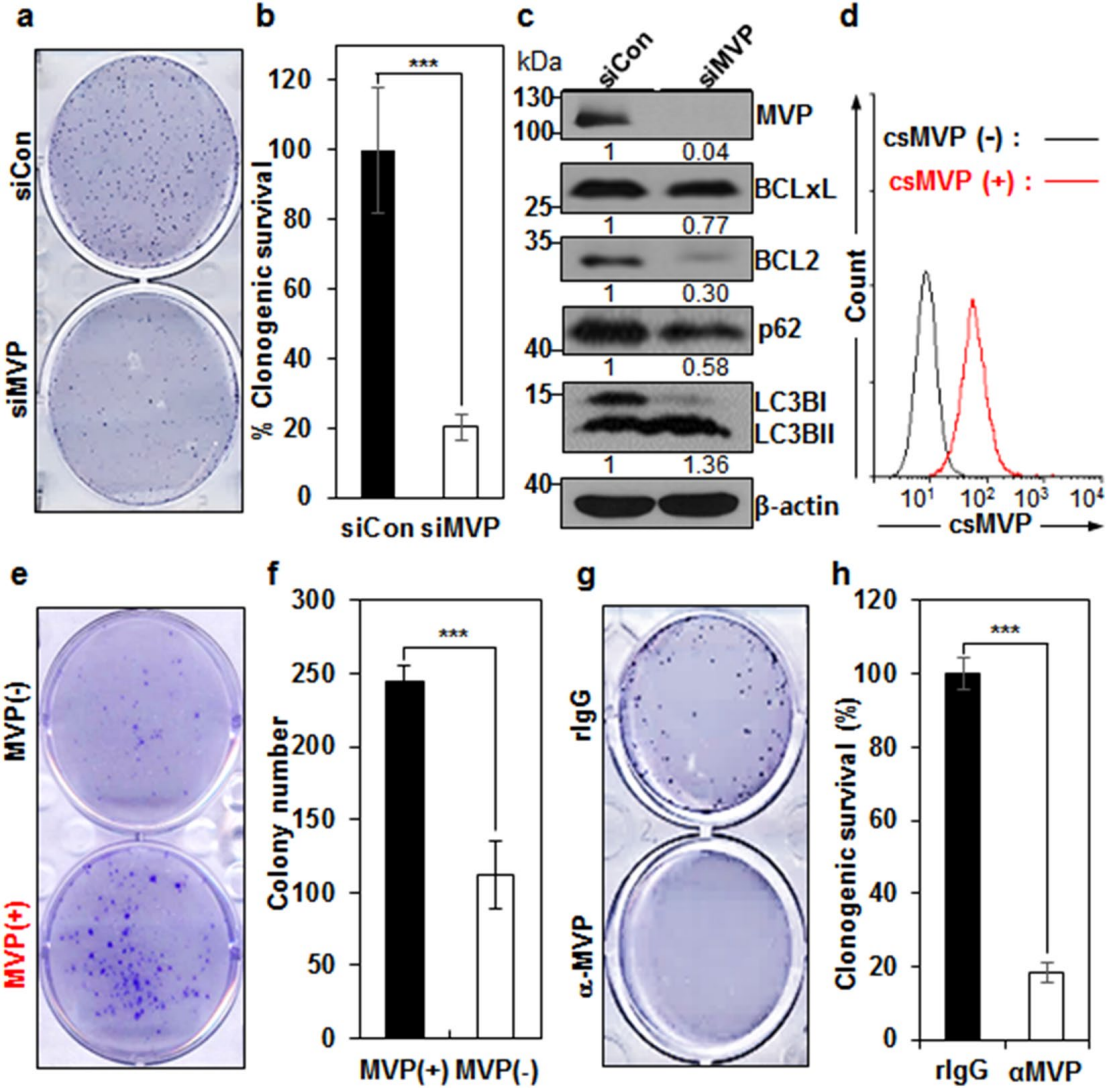
csMVP supports the survival of HCC cells under the stress of low density seeding. (**a**) Decreased clonogenic survival of MVP knockdown Huh7 cells. Clonogenic survival potential was compared between control siRNA (siCon)- and MVP siRNA (siMVP)-treated Huh7 cells. Colonies were stained with crystal violet. (**b**) Graphic presentation of a (*n* = 6). The graphs represent the relative percentage of colony numbers of MVP knockdown Huh7 cells. ***p < 0.005. (**c**) MVP knockdown decreases survival regulators and increases autophagy flux. MVP was knocked down in Huh7 cells, and survival regulators (Bcl-2 and Bcl-xL) and autophagy markers (LC3BI,II and p62) were analyzed by Western blot analysis. Gels were run under the same experimental conditions while images of western blots displayed in cropped format. Full-length blots are presented in Supplementary Fig. 4. (**d**) Cell sorting of csMVP-positive and csMVP-negative Huh7 cells. Shown is flow cytometric validation of the csMVP (+) and csMVP (−) populations after sorting. (**e**) Clonogenic survival assay of csMVP (+) or csMVP (−) Huh7 cells. (**f**) Graph presentation of e. The graphs represent the colony number counted by image analysis software (*n* = 3; ***p < 0.005). (**g**) Decreased clonogenic survival of α-MVP-treated Huh7 cells. Clonogenic survival potential was compared between rIgG- or α-MVP-treated Huh7 cells. Colonies were stained with crystal violet. (**h**) Graphic presentation of g. The graphs represent the relative percentage of colony numbers of α-MVP-treated Huh7 cells (*n* = 3; ***p < 0.005).

The clonogenic assay is a widely used method to determine the survival and growth of cells in cancer cell lines. MVP knockdown decreased the number and size of Huh7 colonies by approximately 80% in the clonogenic survival assay (Fig. 3a,b), demonstrating that MVP enhances HCC cell survival under the stress of low density seeding. To investigate how MVP regulates HCC cell survival, the expression of survival regulators, such as Bcl-2 and Bcl-xL was analyzed in MVP knockdown Huh7 cells. MVP depletion induced decreased expression of Bcl-2 and Bcl-xL in Huh7 cells, which is expected from the previous study^16^ (Fig. 3c and supplementary Fig. 5). MVP depletion also induced autophagy flux by increasing the form of LC3BII and by decreasing survival factor p62 in Huh7 cells (Fig. 3c and supplementary Fig. 5). The results suggest that MVP promotes cell survival in HCC cells by inducing survival factors and inhibiting autophagy flux. To dissect the role of csMVP in HCC cells, csMVP-positive and negative Huh7 cells were sorted and subjected to the clonogenic assay (Fig. 3d). csMVP-positive Huh7 cells showed enhanced clonogenic survival by approximately 2.2-fold, as compared with csMVP-negative Huh7 cells (Fig. 3e,f). Furthermore, treatment of Huh7 cells with α-MVP decreased clonogenic survival of Huh7 cells by approximately 82% (Fig. 3g,h). The results suggest that csMVP supports clonogenic survival of HCC cells when they are exposed to stressful environments.

### csMVP function is mediated through the control of ERK, Akt, mTOR and FAK signaling

**Figure 4:**
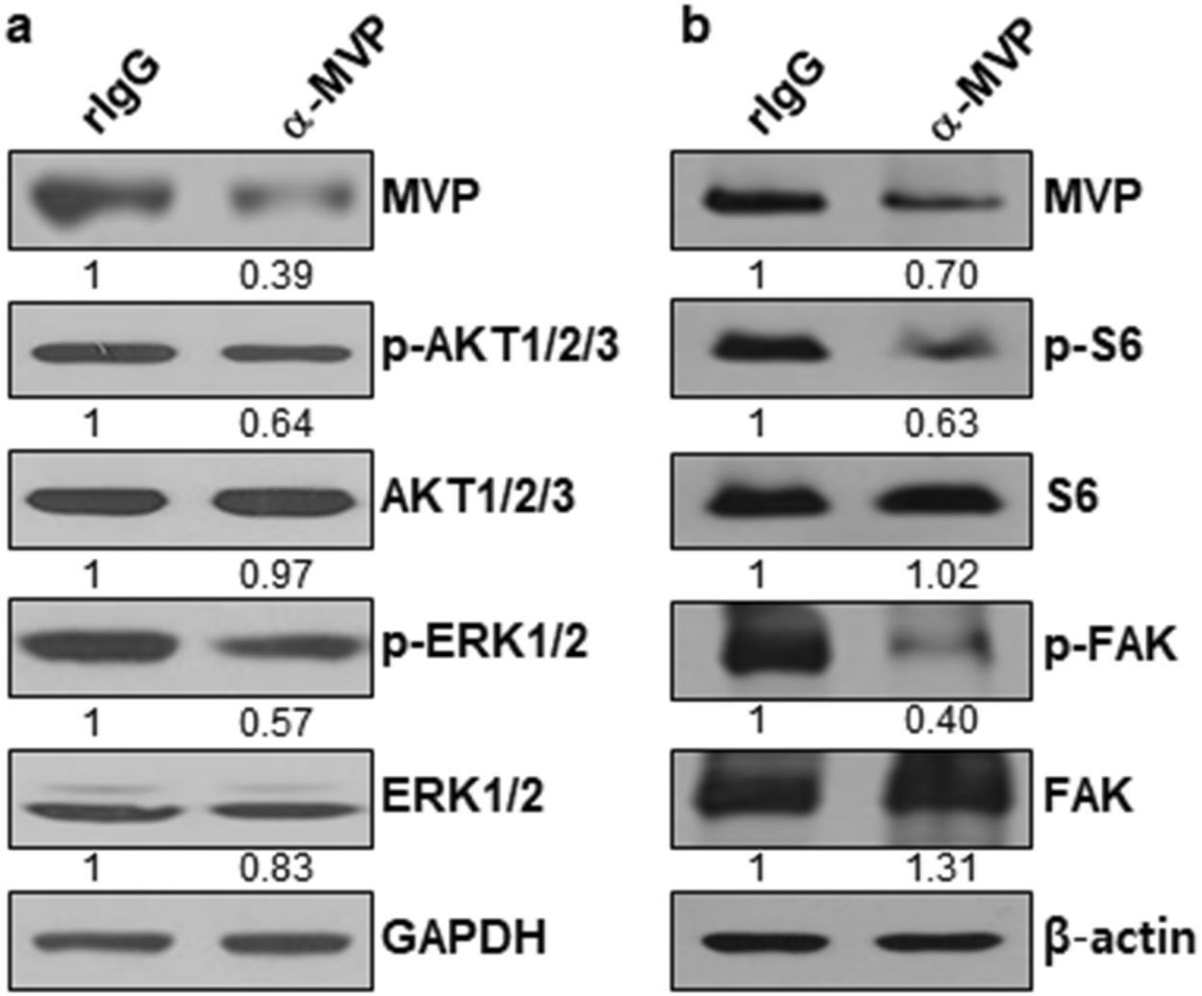
The biological function of csMVP is mediated through the control of mTOR, FAK, ERK and Akt signaling. (**a**) Western blot analysis of MVP, Akt1/2/3 and ERK1/2 in Huh7 cells treated with rIgG or α-MVP. The phosphorylation of Akt1/2/3 and ERK1/2 was also examined in Huh7 cells treated with rIgG or α-MVP. The signal intensities of Western blots were measured quantitatively using the Image J software, and GAPDH was used as a loading control. (**b**) Western blot analysis of MVP, S6, FAK in Huh7 cells treated with rIgG or α-MVP. The phosphorylation of S6 and FAK was also examined in Huh7 cells treated with rIgG or α-MVP. β-actin was used as a loading control.

To further figure out how csMVP promotes cancer growth and metastasis in HCC cells, we analyzed signaling molecules in α-MVP-treated Huh7 cells. Treatment of Huh7 cells with α-MVP reduced MVP protein level by approximately 30–61% (Fig. 4a,b and supplementary Fig. 6). Analysis of the signaling molecules revealed that phosphorylation of Akt1/2/3 and ERK1/2 is decreased in α-MVP-treated Huh7 cells by approximately 36% and 43%, respectively (Fig. 4a and supplementary Fig. 6). Phosphorylation of S6, a downstream effector of mTOR, was also decreased in α-MVP-treated Huh7 cells by approximately 37% (Fig. 4b and supplementary Fig. 6). Phosphorylation of FAK, a key regulator of adhesion and motility in cancer cells, was also decreased in α-MVP-treated Huh7 cells by approximately 60% (Fig. 4b and supplementary Fig. 6). The results suggest that csMVP-associated signaling is mediated through the control of ERK1/2, Akt1/2/3, mTOR and FAK signaling pathways, which are critical for cancer growth, survival and metastasis^21–23^.

### Detection of csMVP-positive CTCs from HCC patients

Based on the finding that csMVP is positively associated with cell proliferation, survival, invasion, and migration in HCC cells, csMVP may serve as a surface marker on long-lived CTCs in patients with HCC. To efficiently detect CTCs in patients with HCC, we depleted CD45-positive cells and analyzed the attached cells on slides, which indicates that only live CTCs are analyzed by α-MVP (Supplementary Fig. 7a). To test the sensitivity and specificity of the method, the increasing number of Huh7 cells was spiked into PBMCs and subjected to CD45 depletion. The number of remaining Huh7 cells was measured with α-MVP. Linear regression of the number of detected Huh7 cells versus the number of Huh7 cells spiked yielded R^2^ of 0.9999, and the average recovery of Huh7 cells was approximately 83% (Supplementary Fig. 7b).

**Figure 5:**
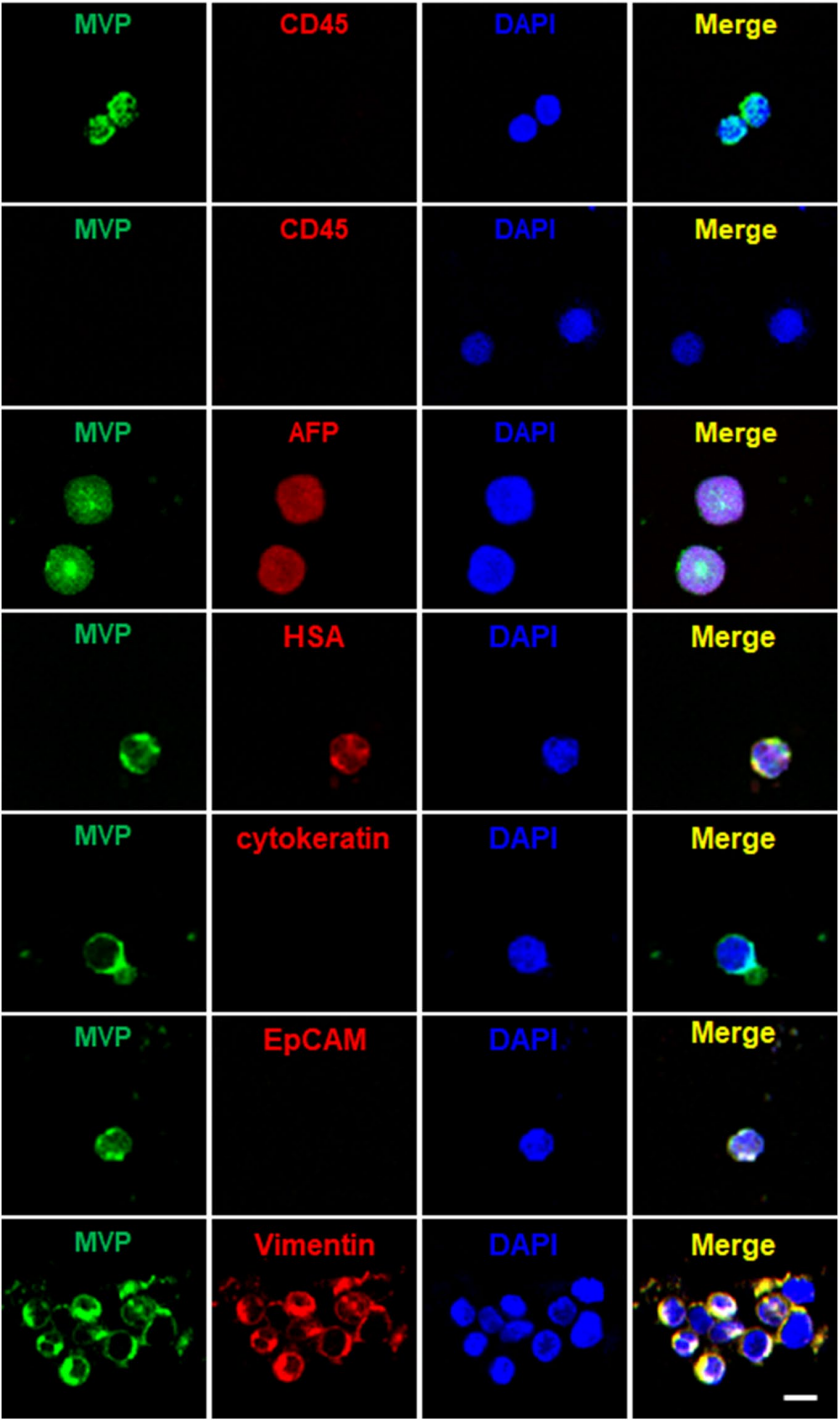
csMVP-positive CTCs show non-epithelial phenotypes, which are CD45-negative, cytokeratin-negative, EpCAM-negative, and vimentin-positive. CD45-depleted CTCs were isolated from HCC patients and stained with α-MVP (green) and anti-CD45 (red) antibodies. For double staining for csMVP and AFP, HSA or cytokeratin, cells were incubated with α-MVP (green), and anti-AFP (red), anti-cytokeratin (red) or anti-HSA (red) antibodies. For double staining for csMVP and EpCAM or vimentin, cells were preincubated with anti-EpCAM (red) or anti-vimentin (red). The cells were then incubated with α-MVP (green). Nuclei were stained with DAPI (blue). The scale bar is 20 μm.

Next, we examined CTCs from 53 HCC, 5 recurrent HCC, and 14 metastatic cancer patients, and also examined blood samples from 2 acute hepatitis patients and 10 healthy volunteers (Table 1 and Supplementary Table 1). No α-MVP-positive CTCs were detected in healthy volunteers and in acute hepatitis patients, indicating a high specificity of csMVP to HCC patients. All csMVP-positive cells from HCC patients were CD45-negative (Fig. 5 and Table 2). Alpha-fetoprotein (AFP) is the well-known gold standard marker of HCC, although the specificity and sensitivity of AFP in HCC is not satisfactory^24–26^. To further examine whether csMVP-positive CTCs are HCC cells, therefore, CTCs from HCC patients were simultaneously stained for csMVP and AFP expression. AFP expression was detected in 27 out of 38 csMVP-positive CTCs (approximately 71%) (Fig. 5 and Table 2). Thus, most of csMVP-positive CTCs are proved to be HCC cells, although the rest of them (29%) are still not identified.

**Table 1:** Cross tabulation of all analyzed patients and healthy volunteers by the number of csMVP-positive CTCs. The number 2.32 is the median number of csMVP-positive CTCs per milliliter (ml) in patients with HCC.

Disease groups	Case No.	No. of csMVP(+) CTC in blood	
		Low (<2.32/ml)	High (≥2.32/ml)
Non-neoplastic group*	**(n = 12)**	**12 (100%)**	**0 (0%)**
Healthy control	(n = 10)	10 (100%)	0 (0%)
Acute hepatitis	(n = 2)	2 (100%)	0 (0%)
Primary HCC*	**(n = 53)**	**28 (52**.**8%)**	**25 (47**.**2%)**
Secondary disease*	**(n = 19)**	**4 (21**.**1%)**	**15 (78**.**9%)**
Recurrent HCC	(n = 5)	1 (20%)	4 (80%)
Metastases to liver	(n = 14)	3 (21.4%)	11 (78.6%)

^*^ *p* < 0.001 by *χ* ^2^-test: non-neoplastic vs. primary HCC, *p* = 0.002; *p*rimary HCC vs. metastatic, *p* = 0.017; non-neoplastic vs. metastatic, *p* < 0.001 by χ^2^-test.

**Table 2:** Expression profile of macrophage and EMT markers in nucleated csMVP^+^ CTCs from HCC patients by double or triple immunofluorescent staining.

Markers	Patients with marker-positive CTCs	Marker-positive CTCs
csMVP^-^DAPI^+^	10/12 (83.3%)	188/372 (50.5%)
CD45^+^	0/12 (0.0%)	0/183 (0.0%)
HSA^+^	2/3 (66. 7%)	10/78 (12.8%)
AFP^+^	1/6 (16.7%)	27/38 (71.1%)
Pan-CK^+^	1/6 (16. 7%)	1/87 (1.2%)
CD68^+^ EpCAM^+^	0/12 (0.0%)	0/82 (0.0%)
CD68^+^ EpCAM^−^	1/12 (8.3%)	1/82 (1.2%)
CD68^−^ EpCAM^+^	0/12 (0.0%)	0/82 (0.0%)
CD68^−^ EpCAM^−^ (csMVP-single-positive)	8/12 (66. 7%)	81/82 (98. 8%)
CD14^+^ Vimentin^+^	0/12 (0. 0%)	0/102 (0. 0%)
CD14^+^ Vimentin^−^	1/12 (8. 3%)	1/102 (1.0%)
CD14^−^ Vimentin^+^	6/12 (50. 0%)	26/102 (25.5%)
CD14^−^ Vimentin^−^ (csMVP single+)	9/12 (75. 0%)	75/102 (73.5%)
Slug^+^ Vimentin^+^	5/9 (55. 6%)	10/135 (7.4%)
Slug^+^ Vimentin^-^	8/9 (88. 9%)	23/135 (17.0%)
Slug^−^ Vimentin^+^	8/9 (88. 9%)	30/135 (22.2%)
Slug^−^Vimentin^−^(csMVP single+)	8/9 (88. 9%)	72/135 (53.3%)
ZEB1^+^ Vimentin^+^	6/9 (66. 7%)	22/163 (13.5%)
ZEB1^+^ Vimentin^−^	7/9 (77. 8%)	13/163 (8.0%)
ZEB1^−^ Vimentin^+^	8/9 (88. 9%)	26/163 (16.05%)
ZEB1^−^Vimentin^−^(csMVP single+)	8/9 (88. 9%)	102/163 (62.6%)
EGFR^+^ Vimentin^+^	0/14 (0. 0%)	0/494 (0.0%)
EGFR^+^ Vimentin^−^	6/14 (42.9%)	36/494 (7.3%)
EGFR^−^Vimentin^+^	12/14 (85.7%)	197/494 (39.9%)
EGFR^−^ Vimentin^−^(csMVP single+)	13/14 (92.9%)	261/494 (52.8%)
EpCAM^+^ Vimentin^+^	0/14 (0. 0%)	0/448 (0.0%)
EpCAM^+^ Vimentin^−^	0/14 (0. 0%)	0/448 (0.0%)
EpCAM^−^ Vimentin^+^	13/14 (92.9%)	218/448 (48.7%)
EpCAM^−^Vimentin^−^(csMVP single +)	13/14 (92.9%)	230/448 (51.3%)

Columns on the right represent the number of patients with CTCs that stain positive for a given marker and number of CTCs scoring positive for each marker. Data are presented as no. (%).

The size of csMVP-positive cells averaged 20 μm with a range between 12.3 and 39.6 μm. (Supplementary Table 2). Among all DAPI-positive and CD45-negative cells (average size 20 μm), α-MVP was able to detect approximately 51% of them (the second row in Fig. 5 and Table 2), suggesting that csMVP-positive CTCs are approximately half of all putative CTCs in HCC patients. When the expression of hepatocyte specific antigen (HSA) was examined in the csMVP-positive CTCs, approximately 13% of csMVP-positive cells were HSA-positive (Fig. 5 and Table 2). When the expression of cytokeratin was examined with an anti-cytokeratin antibody, cytokeratin was not detected in csMVP-positive CTCs, except for one large-sized CTC (approximately 40 μm) (Fig. 5 and Supplementary Fig. 8a). The result suggests that csMVP-positive CTCs show almost nonepithelial phenotype. When the expression of EpCAM was examined in csMVP-positive CTCs, all of the csMVP-positive cells were EpCAM-negative (Fig. 5 and Table 2). Instead, a considerable number of csMVP-positive CTCs were vimentin-positive (Fig. 5 and Table 2), suggesting that csMVP-positive CTCs may show nonepithelial phenotype.

**Figure 6:**
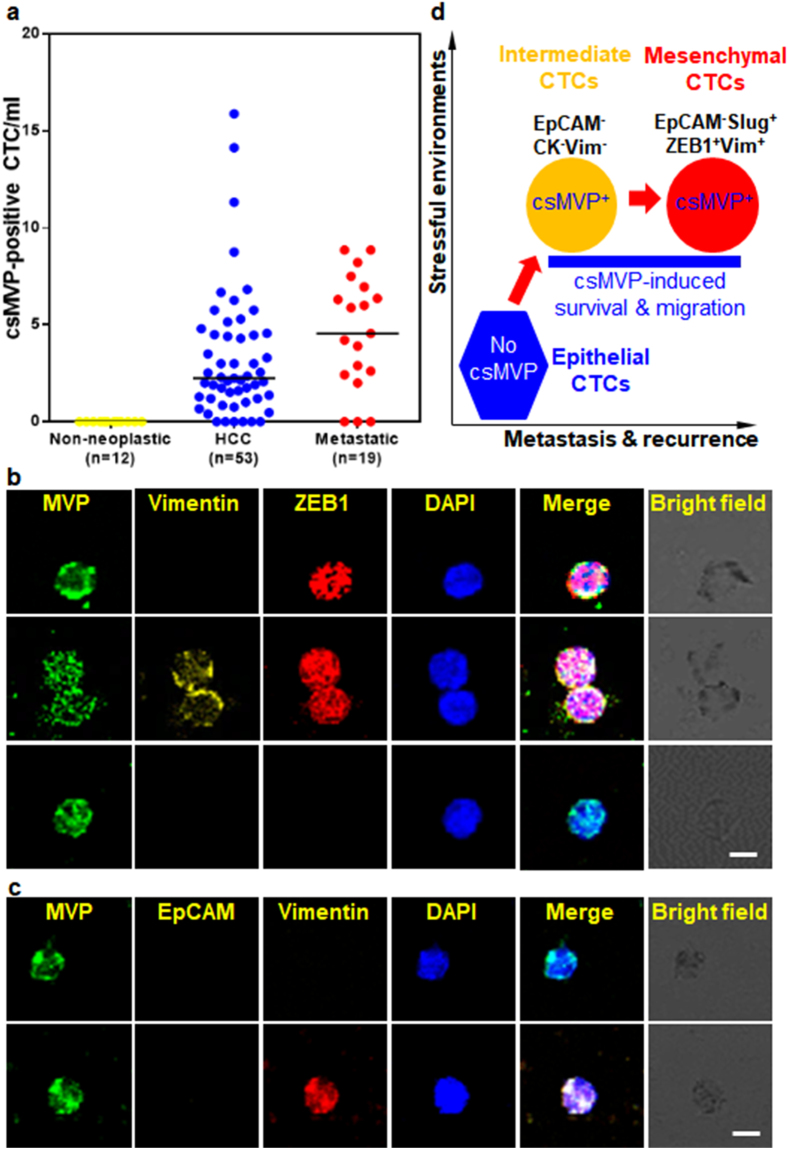
csMVP-positive CTCs are increased in patients with metastatic cancers and show mesenchymal phenotype and intermediate phenotype with neither epithelial nor mesenchymal markers. (**a**) csMVP-positive CTCs are detected in HCC patients, but not healthy donors and hepatitis patients, and is further increased in patients with metastatic cancers. The number of csMVP-positive CTCs were enumerated in patients with non-neoplastic tumor (healthy volunteers and hepatitis patients), HCC, or metastatic cancers (recurrent HCC and metastatic cancer). Horizontal bar in the graph represents the median value of the csMVP-positive CTCs. (**b**) csMVP-positive CTCs show EMT phenotype with ZEB1 expression. CD45-depleted CTCs were incubated with α-MVP (green), anti-vimentin (yellow), and anti-ZEB1 (red) antibodies. Nuclei were stained with DAPI (blue). The scale bar is 20 μm. (**c**) CD45-depleted CTCs were incubated with anti-vimentin (red), α-MVP (green), and anti-EpCAM (yellow) antibodies. Nuclei were stained with DAPI (blue). The scale bar is 20 μm. (**d**) Proposed model for the role of csMVP in cancer metastasis and recurrence.

csMVP-positive CTCs were detected in 52 out of 58 HCC patients (89.7%), and the cell number isolated from these HCC patients ranged between 0.4 and 15.9 per ml (median number 2.32 CTCs/ml) (Fig. 6a and Table 1). Although the number of csMVP-positive CTCs was higher in HCC patients than healthy volunteers, there was no statistically significant correlation between the number of csMVP-positive CTCs and clinicopathologic factors (Supplementary Table 3). However, the number of csMVP-positive CTCs was tended to be higher in patients with advanced tumor stage group (Supplementary Table 3). Furthermore, the number of csMVP-positive CTCs was tended to be higher in patients with recurrent HCC or hepatic metastasis of other cancer groups, compared to primary HCC group (Fig. 6a and Table 1) (*p* < 0.017). The results show that the number of csMVP-positive CTCs is significantly increased according to progression of tumor. Furthermore, the number of csMVP-positive CTCs was significantly decreased after surgery (*p* value = 0.027), although the number of patients was low (Supplementary Table 4). Again, the results suggest that the number of csMVP-positive CTCs is closely associated with progression of tumor in HCC patients.

### csMVP-positive CTCs do not have macrophage phenotype

A recent study reported that circulating giant macrophages are a potential biomarker of solid tumors^27^. The macrophage markers CD68 and CD14 are positive for the circulating endothelial cells in patients with gynecologic cancers^28^. In the csMVP/CD68/EpCAM staining, there were no EpCAM-positive CTCs among csMVP-positive CTCs (Table 2). Only 1 out of 82 csMVP-positive CTCs was positive for CD68 (Supplementary Fig. 8b and Table 2). In the csMVP/CD14/vimentin staining, only 1 out of 102 csMVP-positive CTCs was positive for CD14 (Supplementary Fig. 8c and Table 2). Based on the size and morphology, furthermore, the rarely detected cells seemed to be real circulating macrophages which were not depleted during the depletion process of CD45-positive cells. Thus, the expression of macrophage markers was hardly detected in csMVP-positive CTCs. Most of CTCs were csMVP-single positive, and 25.5% of csMVP-positive CTCs were vimentin-positive (Table 2). The results suggest that csMVP-positive CTCs do not belong to the circulating cells with macrophage phenotype.

### csMVP-positive CTCs show mesenchymal phenotype or intermediate phenotype with neither epithelial nor mesenchymal markers

To examine the characteristics of csMVP-positive CTCs in terms of the expression of mesenchymal markers, triple staining for csMVP/Slug/vimentin and csMVP/ZEB1/vimentin was further performed. In the csMVP/Slug/vimentin staining, 46.7% of csMVP-positive CTCs were positive for the mesenchymal markers Slug or vimentin, while 53.3% of csMVP-positive CTCs were csMVP-single positive (Supplementary Fig. 9a and Table 2). In the csMVP/ZEB1/vimentin staining, 37.4% of csMVP-positive CTCs were positive for the mesenchymal markers ZEB1 or vimentin, while 62.6 of csMVP-positive CTCs were csMVP-single positive (Fig. 6b and Table 2). The results suggest that 37–47% of csMVP-positive CTCs were CTCs with mesenchymal phenotype depending on the experimental setting, while 53–63% of csMVP-positive CTCs were CTCs with neither epithelial nor mesenchymal phenotype.

EGFR expression also promotes EMT-like transition and cancer cell survival with subsequent Twist, Snail, and Slug induction^29–31^. In the csMVP/EGFR/vimentin staining, approximately 47.2% of csMVP-positive CTCs were positive for EGFR or vimentin, while approximately 52.8% of csMVP-positive CTCs were just csMVP-single positive (Table 2 and supplementary Fig. 9b). Interestingly, approximately 7.3% of csMVP-positive CTCs were EGFR-positive and vimentin-negative, suggesting that they may be the intermediate type before becoming the fully developed mesenchymal phenotype. In the csMVP/EpCAM/vimentin staining, csMVP/EpCAM double positive CTCs were 0% among 448 csMVP-positive CTCs (Fig. 6c and Table 2), strongly suggesting again that csMVP-positive CTCs show nonepithelial phenotype. Among 448 csMVP-positive CTCs, csMVP/vimentin double positive CTCs were 218 (48.7%). Approximately 51% of csMVP-positive CTCs were csMVP-single positive, which did not express both EpCAM and vimentin. Overall, approximately 25.5–48.7% of csMVP-positive CTCs were positive for the mesenchymal markers Slug, ZEB1, EGFR, or vimentin, while the rest of them (51.3–73.5%) were just csMVP-single positive (Table 2). Thus, the results suggest that csMVP-positive CTCs consist of mesenchymal phenotype and intermediate phenotype with neither epithelial nor mesenchymal markers in HCC patients.

## Discussion

The present study demonstrated, for the first time, that MVP is expressed on the cell surface. To figure out how MVP translocates to the cell surface, we treated Huh7 cells with some chemical inhibitors of cancer cell signaling pathways, and found that U0126 (ERK inhibitor) and rapamycin (mTOR inhibitor) obviously decreased cell surface expression of MVP (Supplementary Fig. 1b). Treatment of Huh7 cells with α-MVP recognizing csMVP decreased MVP protein level and decreased phosphorylation of ERK1/2 and S6, a downstream effector of mTOR (Fig. 4a,b and supplementary Fig. 6). The results suggest that cell surface translocation of MVP may be closely associated with ERK and mTOR signaling. A recent study suggests that MVP is found in exosomes and involved in exporting miR-193a via exosomes during colon cancer metastasis to the liver^32^. The study suggests that MVP may exploit the exosomal pathway to move to the cell surface. How MVP is translocated to the cell surface is the interesting next subject to study.

Cell surface expression of MVP were observed in HCC cell lines and some other cancer cell lines, although it was not detected on PBMCs and primary hepatocytes (Fig. 1). Studies have shown that stress-inducible MVP expression plays an important role in preventing apoptosis induction^15,16,33,34^. Previous studies led us to speculate that some of the MVP protein could be translocated to the cell surface when the cells are exposed to stressful environments. Serum starvation, DNA damage, and detachment stress induced cell surface expression of MVP in Huh7 and A549 cells (Fig. 1c,d and Supplementary Fig. 2). Cell sorting also demonstrated that csMVP-positive HCC cells have much higher clonogenic survival than csMVP-negative HCC cells (Fig. 3e,f). Treatment with α-MVP also inhibited the clonogenic survival of HCC cells (Fig. 3g,h). csMVP also supported the migratory and invasive potential of HCC cells (Fig. 2d–g). Thus, csMVP contributes to the survival and invasive and migratory potential of HCC cells. The acquisition of invasive and migratory properties and survival capacity is critical for the generation and maintenance of CTCs during tumor progression and metastasis. Therefore, we postulated that the expression of csMVP would be beneficial for the generation and maintenance of CTCs in HCC patients, where CTCs are scarcely detected by anti-EpCAM antibody. Actually, csMVP-positive CTCs were efficiently (89.7%) detected in HCC patients, and the number of csMVP-positive CTCs was further increased in patients with metastatic cancers. Interestingly, all csMVP-positive CTCs show mesenchymal phenotype or intermediate phenotype with neither epithelial nor mesenchymal markers, suggesting that csMVP is a novel surface marker for CTCs with nonepithelial phenotype.

EpCAM and cytokeratin expression was hardly detected in csMVP-positive CTCs, suggesting that csMVP-positive CTCs do not belong to epithelial phenotype. Instead, 25.5–48.7% of total csMVP-positive CTCs are positive for the expression of vimentin, Slug, or ZEB1 in HCC patients (Table 2), indicating that a substantial number of the csMVP-positive CTCs have mesenchymal phenotype. The results were expected from the finding that csMVP positively regulates HCC cell migration and invasion *in vitro* (Fig. 2d–f). Vimentin, ZEB1, and Slug genes linked with the EMT process are essential for tumor cells to exhibit survival capacity^6^. Therefore, it is tempting to speculate that EMT-associated survival signals contribute to the existence of csMVP-positive CTC with EMT phenotype. However, the rest of the csMVP-positive CTCs (51.3–73.5%) were vimentin-, Slug- or ZEB1-negative (Table 2), suggesting that they belong to the intermediate phenotype with neither epithelial nor mesenchymal markers. CTCs with intermediate phenotype are considered rare and temporary because the cells are rarely detected^35^. Therefore, csMVP-associated pro-survival signals may result in the high detection rate of intermediate CTCs in HCC patients. It is also tempting to speculate that the intermediate CTCs with csMVP-associated pro-survival signals have metastable phenotype and live longer even under stressful environments. csMVP-positive intermediate CTCs may also have a chance to become EMT-phenotypic CTCs, which provide some positive effects on cancer progression in HCC patients. Thus, csMVP-positive CTCs may have a chance to become hibernating cells in liver or other tissues, and may cause a high incidence of recurrent metastatic HCCs because of their long-lasting survival capacity. Actually, csMVP-positive CTCs were increased in CRC patients with liver metastases, although their primary cancers were removed in these patients (Fig. 6a and Table 1). A proposed model for the function of csMVP in HCC metastasis and recurrence is presented (Fig. 6d). Further investigation in a larger cohort of metastatic and recurrent HCC patients could verify the above interpretations.

Generally, the number of CTCs is lower in HCC patients than in other cancer patients, when the EpCAM-based CTC detection system is used to measure the number of CTCs^9–11,36^. Two studies reported that EpCAM-positive CTCs are detected in 41% and 40% (>0.26 CTCs/ml blood) of HCC patients^10,36^. Other two studies also showed that EpCAM-positive CTCs are detected in 28% and 31% (>0.13 CTCs/ml blood) of HCC patients^9,11^. Successful metastasis may depend on the generation of semi-mesenchymal or mesenchymal CTCs during cancer metastasis^37,38^. Actually, several studies have shown a close correlation between EMT-marker expression of CTCs and cancer metastasis in breast, prostate, and colon cancers^39–43^. The results suggest the possibility that nonepithelial CTCs may be mainly associated with HCC cancer progression. Therefore, some studies have attempted to identify novel surface markers on CTCs in patients with HCC. Asialoglycoprotein receptor (ASGPR) was used to detect CTCs in HCC patients, and CTCs were identified in 81–89% of HCC patients (≥0.2 CTCs/ml blood), respectively^44,45^. Here, csMVP-based analysis of CTCs also shows high efficiency (89.7%, ≥0.4–15.9 CTCs/ml blood) to isolate and characterize CTCs from HCC patients. Furthermore, csMVP-positive CTCs show EMT phenotype, which is not detectable in the EpCAM-based CTC detection system. csMVP-positive CTCs also show their unique intermediate phenotypes which are not detectable even in the ASGPR-based CTC detection system. Furthermore, csMVP may be universally an important molecule on metastatic CTCs from various human cancers because csMVP-positive CTCs were detected in patients with liver, colorectal, lung, and stomach cancer (Supplementary Table 1).

In conclusion, we found that MVP is expressed on the surface of HCC cells but not on normal hepatocytes. csMVP is induced under stressful environments and contributes to the proliferation, clonogenic survival, and migratory and invasive capacity of HCC cells. Thus, csMVP-positive HCC cells may be a subpopulation that survives longer under stressful environments. The function of csMVP is mediated through mTOR, FAK, ERK and Akt signaling pathways, which are closely associated with cancer cell survival and metastasis. csMVP-positive CTCs are efficiently (89.7%) detected in patients with HCC but not in healthy volunteers. The number of csMVP-positive CTCs is further increased in patients with metastatic cancers. Almost all csMVP-positive CTCs are CD45-, EpCAM-, and cytokeratin-negative, suggesting that they are CTCs with nonepithelial phenotype. More than half of the csMVP-positive CTCs (51.3–73.5%) show intermediate phenotype with neither epithelial nor mesenchymal markers, while the rest of them show mesenchymal phenotypes with vimentin, Slug, or ZEB1 expression. The results suggest that csMVP promotes cancer progression and serves as a novel surface biomarker for detecting and evaluating mesenchymal and intermediate CTCs in patients with HCC and metastatic cancers.

## Methods

### Cells, tumor sphere culture, and drug treatment

HCC cell lines (Huh7 and HepG2) and human nonsmall cell lung carcinoma cell (NSCLC) lines (A549 and H358) were purchased from Korean Cell Line Bank (Seoul, Korea). Human immortalized liver epithelial cell line (THLE2) and human primary hepatocyte were purchased from the American Type Culture Collection (Manassas, USA) and Thermo Fisher Scientific (Waltham, USA), respectively. For the sphere culture, single cell suspensions of Huh7 cells were suspended in DMEM/F12 medium supplemented with B27 supplement (Life Technologies, Seoul, Korea), 0.01% antibiotic-antimycotic (Life Technologies), 20 ng/mL EGF (Peprotech, Seoul, Korea), and 20 ng/mL basic fibroblast growth factor (R&D systems, Minneapolis, USA), and were seeded into ultra-low attachment 6-well plates (Corning, Seoul, Korea). For the anti-cancer drug treatment, Huh7 and A549 cells were treated with dimethyl sulfoxide (DMSO) or 2 µM of cisplatin (Sigma-Aldrich, Seoul, Korea), or Etoposide (Sigma-Aldrich) in RPMI1640 medium for 4 days. For the analysis of cell surface expression of MVP, Huh7 cells were treated with DMSO, 50 μM LY294002, 20 μM U0126 or 10 μM rapamycin (all from Sigma-Aldrich) in medium for 48 hrs.

### Patients and sample collection

From June 2014 to December 2016, 48 HCC, 5 recurrent HCC, and 13 metastatic cancer patients undergoing curative resection were recruited into a prospective study. Peripheral blood samples (approximately 10 ml) were obtained in heparin-containing collection tubes with informed consent from patients who would undergo curative hepatectomy in the Department of Surgery, Samsung Medical Center (Seoul, Korea). Peripheral blood samples were also obtained from 2 acute hepatitis patients and 10 healthy volunteers with the same informed consent. Blood samples were divided depending on the demands for multiple staining. Cancer staging by the modified Union for International Cancer Control (mUICC, 2000) and the American Joint Committee on Cancer (AJCC, 2010) were used. For the experiments involving human samples, approval was obtained from the institutional review board of Samsung Medical Center (SMC IRB No.2015–10–029). All subsequent experiments were also performed in accordance with the relevant laws and institutional guidelines.

### Antibody biotinylation

Antibody was biotinylated by using DSB-X biotin labeling kit (Life Technologies) according to manufacturer’s procedure.

### Enrichment of CTCs by depletion of CD45-postive cells

Cells were enriched from blood samples within 6 hours after collection. Peripheral blood mononuclear cells (PBMC) were separated from peripheral blood of healthy donors or patients by Ficoll-Paque Plus (GE Healthcare, Seoul, Korea) gradient centrifugation. PBMCs were washed with phosphate buffered saline (PBS, pH 7.4) containing 0.5% bovine serum albumin and 2 mM EDTA, and resuspended in the same buffer at a concentration of 1 × 10^8^ cells/mL. The enrichment of CTCs by CD45 depletion of the leukocyte fraction was performed using the Human CD45 Depletion Kit (Stem Cells Technologies, Vancouver, Canada). The recovered cells were centrifuged at 3560 × g for 5 minutes, resuspended in 400 μl of RPMI1640 medium, and seeded onto glass slides coated with 0.1 μg/ml of poly-L-lysine. To induce the spontaneous binding of the live cells to the glass slides, the cells were incubated for 2–4 hours at room temperature (RT). Unbound cells were washed with PBS before fixation. Bound cells were fixed in 3.7% paraformaldehyde (PFA) and stored in refrigerator for further use.

### Immunocytochemistry

Fixed Huh7 cells were blocked with 10% normal horse serum and then incubated with rabbit polyclonal anti-MVP against the C-terminal region of MVP (α-MVP, Aviva, San Diego, USA) and mouse monoclonal anti-EGFR (Santa Cruz Biotechnology, Santa Cruz, USA). The cells were further incubated with Dylight 650-conjugated anti-rabbit IgG (Thermo Fisher Scientific) and Alexa 488-conjugated-anti-mouse IgG (Life Technologies). Nuclei were labelled with 4′,6-diamidino-2-phenylindole (DAPI).

For immunostaining for CTCs from blood samples, CD45-depleted cells were seeded on poly-L-lysine coated glass as described above. For double immunofluorescence staining for MVP and CD45, cells were fixed in 3.7% PFA, blocked with 10% normal horse serum, and then incubated with α-MVP (Aviva systems biology) and mouse monoclonal anti-CD45 (Stem Cell Technologies) antibodies. The cells were further incubated with Alexa 488-conjugated anti-rabbit IgG (Life technologies) and Dylight 649-conjugated mouse IgG (Vector laboratories, Burlingame, USA). For double immunofluorescence staining for MVP and cytokeratin or HSA, CD45-depleted cells were incubated with biotin-conjugated α-MVP and Dylight 488-conjugated streptavidin (Vector Laboratories). The cells were then incubated with phycoerithrin (PE)-conjugated anti-cytokeratin (BD Biosciences, Seoul, Korea) or Dylight 650-conjugated HSA (Novus Biologicals, Littleton, USA). For double immunofluorescence staining for MVP and EpCAM, vimentin, or AFP, cells preincubated with rabbit polyclonal anti-EpCAM, anti-vimentin (Santa Cruz Biotechnology), or AFP (AbCAM) were incubated with Dylight 650-conjugated anti-rabbit IgG (Thermo Fischer Scientific). The cells were incubated with biotin-conjugated α-MVP and further incubated with Dylight 488-conjugated streptavidin (Vector Laboratories).

For triple immunofluorescence staining for MVP, vimentin or EpCAM, and CD14 or CD68, CD45-depleted cells were first incubated with biotin-conjugated α-MVP (Aviva systems biology) and rabbit polyclonal anti-vimentin or anti-EpCAM (Santa Cruz Biotechnology). The cells were further incubated with Dylight 488-conjugated streptavidin (Vector Laboratories), Dylight 650-conjugated-anti-rabbit IgG (Thermo Fisher scientific) and PE-conjugated anti-CD14 or anti-CD68 (MACS, Seoul, Korea). For triple immunofluorescence staining for MVP, vimentin and EGFR, CD45-depleted cells were fixed 3.7% PFA, permeabilized with 0.1% Triton X-100, blocked with 10% normal horse serum, and then incubated with rabbit polyclonal anti-EGFR (Santa Cruz Biotechnology) and Dylight 650-conjugated rabbit IgG (Thermo Fisher Scientific). The cells were incubated with biotin-conjugated α-MVP and Dylight 488-conjugated streptavidin (Vector Laboratories). The cells were further incubated with Alexa 555-conjugated anti-vimentin (Cell Signaling Technology). For triple immunofluorescence staining for MVP, EpCAM and vimentin, CD45-depleted cells were fixed, permeabilized, blocked and incubated with rabbit polyclonal anti-vimentin (Santa Cruz Biotechnology) and Dylight 650-conjugated rabbit IgG (Thermo Fisher Scientific). The cells were incubated with biotin-conjugated α-MVP and Dylight488-conjugated streptavidin (Vector Laboratories). The cells were further incubated with Alexa 555-conjugated anti-EpCAM (Cell Signaling Technology). For triple immunofluorescence staining for MVP, vimentin, and Slug or ZEB1, CD45-depleted cells were first incubated with α-MVP (Aviva systems biology) and Alexa 488-conjugated rabbit IgG (Life technologies) after fixation, permeabilization and blocking. The cells were further incubated with Alexa 555-conjugated anti-vimentin (Cell Signaling Technology) and Alexa 647-conjugated anti-Slug or anti-ZEB1 antibodies. Between each step, cells were four times washed with PBS containing Ca^2+^ and Mg^2+^. Nuclei were stained with DAPI. Fluorescence signals were detected with a Leica TCS SP5 confocal microscope. The quantitative number of CTCs was expressed as the number of CTCs per 1 ml (or 7.5 ml) of blood. All samples were processed in a blinded manner and result interpretation was also independently accomplished by specialists specifically trained and with no information about the clinical status of patients.

### Spiking assay

To demonstrate the specificity and reproducibility of CTC detection by α-MVP, 10, 30, 50, and 100 Huh7 cells were spiked into 10 million PBMCs from healthy donors. After depletion of CD45-positive cells, the remaining cells were seeded onto poly-L-lysine coated slides, fixed, and stained with Dylight 650-conjugated anti-rabbit IgG. Between each step, cells were washed with PBS containing Ca^2+^- and Mg^2+^. The same experiments were performed in triplicates, and fluorescence signals were detected with a Leica TCS SP5 confocal microscope.

### Cell surface biotinylation, immunoprecipitation, and Western Blotting

Cell surface biotinylation, immunoprecipitation, and Western Blotting were performed as described previously^46–48^. The primary antibodies used were as follows: α-MVP (Abcam, Cambridge, UK), rabbit monoclonal antibody against EGFR (Leica Biosystems, Newcastle, UK), rabbit polyclonal antibodies against Bcl-2, Bcl-xL (all from Santa Cruz Biotechnology), p62, E-cadherin (all from Abcam), and LC3B (Novus Biologicals). Antibody binding was visualized with ECL Western Blotting Detection Reagents (GE Healthcare). The signal intensities of Western Blots were measured quantitatively using the ImageJ.

### Western Blotting of signaling molecules

Western blotting was performed as described previously^46,47^. The primary antibodies used were as follows: rabbit polyclonal antibodies against MVP (AbCAM), rabbit polyclonal antibodies against FAK, ERK1/2, Akt1/2/3, phospho-Akt1/2/3, (all from Santa Cruz Biotechnology), phospho-FAK (Tyr397), phospho-ERK1/2 (T202/Y204), S6, phospho-S6 (Ser240/244) (all from Cell Signaling Technology). For Western blot analysis, Huh7 cells were lysed with RIPA buffer (1% NP40, 150 mM NaCl, 50 mM Tris-HCl, pH 7.4, 0.1% SDS, and 0.5% Deoxycholate)

### *In vitro* cell invasion and migration assays

For invasion assays, the upper and lower sides of the membranes in Trans-well chambers (Corning) were coated with Matrigel (BD Biosciences). Huh7 cells were suspended (2.0 × 10^4^ cells/mL) in serum-free medium with 50 μg/mL of rabbit IgG isotype control (rIgG) or α-MVP and placed on the top of the chamber. DMEM medium containing 10% fetal bovine serum (FBS) was placed in the lower chamber. After incubation for 24 to 72 hours, the cells that invaded to the lower surface of the filters were fixed and stained with crystal violet (Sigma-Aldrich). For migration assays, the same chambers were used without Matrigel coating. All the following experiments were the same as the above assay. Crystal violet stained cells were analyzed by Cell Counter program.

### siRNAs

Small interfering (siRNA) oligonucleotides targeting MVP (Santa Cruz Biotechnology) were used with All Stars negative control siRNA (Qiagen, Valencia, USA). Cells were transfected with 100 nM control or MVP siRNA by RNAimax (Life Technologies). The cells were transfected the second time after 48 hours following the first transfection and then incubated for additional 24 hours before harvesting for analysis.

### Flow cytometry and cell sorting

Flow cytometry was performed using α-MVP as described previously^46,47^. For isolation of csMVP-positive and -negative cells, 5 × 10^6^ Huh7 cells were first incubated for 30 minutes at 4 °C in the dark with 20 μg/ml α-MVP. The cells were washed with PBS supplemented with 5% FBS and sorted by BD FACSAria II (BD Biosciences).

### Apoptosis and cell viability assay

To detect apoptosis, control and MVP knockdown Huh7 cells were cultured for 3 days and stained with PI and Fluorescein isothiocyanate (FITC)-conjugated annexin V (BD Biosciences) according to the manufacturer’s protocol. To measure cell viability, Huh7 cells were transfected with control or MVP siRNA. To measure cell viability in the presence of α-MVP antibodies, Huh7 or HepG2 cells were treated with 50 μg/ml of rIgG or α-MVP antibodies for 2 days. Antibodies containing sodium azide were dialyzed against PBS before use. Viable cell numbers were determined by Trypan Blue exclusion assay.

### Clonogenic survival assay

To determine clonogenic survival of MVP knockdown Huh7 cells, control or MVP siRNA-transfected Huh7 cells were harvested after 2 days of siRNA transfection. Cells (1 × 10^4^) were plated on 6-well plate with complete medium. After 7 days of incubation, the cells were stained with crystal violet (Sigma-Aldrich), and their colonies were counted. Huh7 cells (1 × 10^4^ cells) were also treated with 20 μg/ml of rIgG or α-MVP in 6-well plates for 7 days, and they were stained with crystal violet before measurement by Cell Counter program.

### Statistical analysis

Statistical analyses were performed with SPSS ver. 22 (IBM Co., Armonk, USA). The independent sample t-test was used to compare the numbers of csMVP-positive CTCs/ml in healthy volunteers and patients with HCC, recurrent HCC, and metastatic cancers. The χ^2^-test was used to evaluate the relationship between the presence of csMVP-positive CTCs and each clinicopathological parameters in HCC patients. A *p*-value of less than 0.05 was considered statistically significant.

## Electronic supplementary material

supplementary Figures 1–9

supplementary Table 1

supplementary Table 2

supplementary Table 3

supplementary Table 4
