# Supplementary material for: Cell-surface major vault protein promotes cancer progression through harboring mesenchymal and intermediate circulating tumor cells in hepatocellular carcinomas
Source: Sci Rep. 2017 Oct 16;7:13201. doi: 10.1038/s41598-017-13501-1 (PMC5643512; doi:10.1038/s41598-017-13501-1)
Supplement: Supplementary file 1 — supplementary Figures 1–9 [file 41598_2017_13501_MOESM1_ESM.pdf]

Cell-surface major vault protein promotes cancer progression through harboring mesenchymal and intermediate circulating tumor cells in hepatocellular carcinomas

Hyun Min Lee[1*], Jae Won Joh[2*], Se-Ri Seo[1], Won-Tae Kim[1], Min Kyu Kim[1], Hong Seo Choi[1], So Young Kim[1], Young-Joo Jang[3], Dong Hyun Sinn[4], Gyu Seong Choi[2], Jong Man Kim[2], Choon Hyuck David Kwon[2], Hee Jin Chang[5], Dae Shick Kim[6], Chun Jeih Ryu[1]

[1]Department of Integrative Bioscience and Biotechnology, Institute of Anticancer Medicine Development, Sejong University, Seoul, Korea.

[2]Department of Surgery, Samsung Medical Center, Sungkyunkwan University School of Medicine, Seoul, Korea.

[3]Department of Nanobiomedical Science, BK21 PLUS Global Research Center for Regenerative Medicine, Dankook University, Cheonan, Korea.

[4]Department of Medicine, Samsung Medical Center, Sungkyunkwan University School of Medicine, Seoul, Korea.

[5]Center for Colorectal Cancer, Research Institute and Hospital of National Cancer Center, Goyang-si, Korea.

[6]Department of Pathology, Samsung Medical Center, Sungkyunkwan University School of Medicine, Seoul, Korea.

*These authors contributed equally to this work.

Correspondence and requests for materials should be addressed to C.J.R (email:cjryu@sejong.ac.kr) or to D.S.K(email:oncorkim@skku.edu).

Supplementary Figure 1

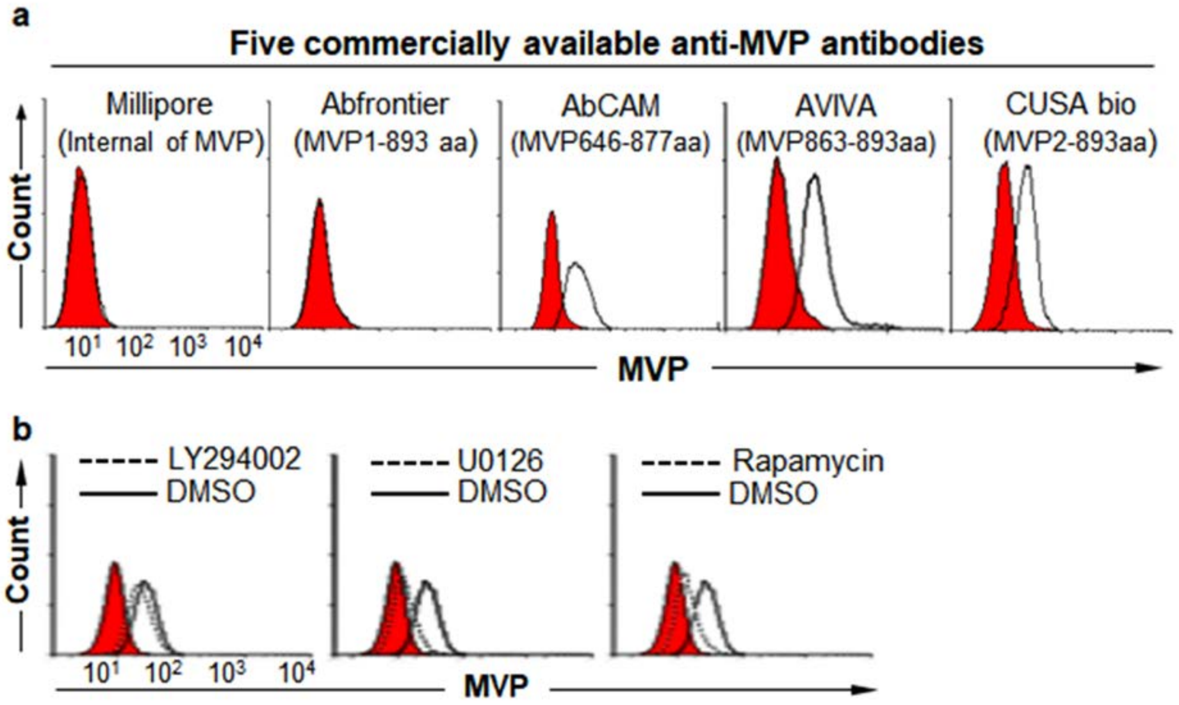

Supplementary Figure 1. csMVP is detected in Huh7 cells with some anti-MVP antibodies, and is downregulated in the presence of U0126 and rapamycin. (a) Flow cytometry analysis of Huh7 cells with 5 different anti-MVP antibodies. (b) Flow cytometry analysis of Huh7 cells with anti-MVP antibody in the presence of dimethyl sulfoxide (DMSO), LY294002 (Akt inhibitor), U0126 (ERK inhibitor), and rapamycin (mTOR inhibitor).

Supplementary Figure 2

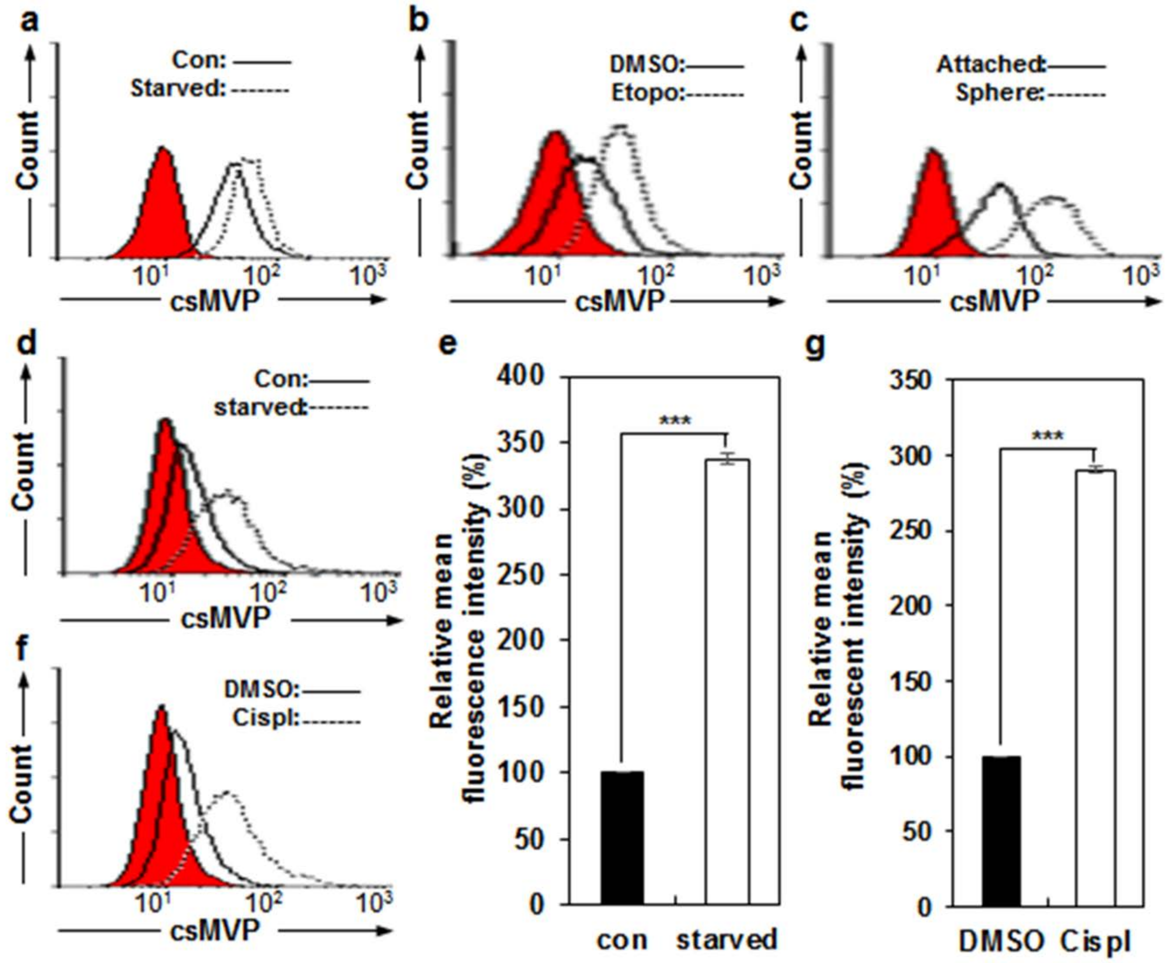

Supplementary Figure 2. Expression of csMVP is induced under stressful conditions. (a) Induced expression of csMVP on the surface of serum-starved Huh7 cells. Huh7 cells were serum-starved and analyzed for the expression of csMVP by flow cytometric analysis with α-MVP. (b) Induced expression of csMVP on the surface of etoposide-treated Huh7 cells. Huh7 cells were treated with DMSO or etoposide (Etopo) and analyzed for the expression of csMVP by flow cytometric analysis with α-MVP. (c) Induced expression of csMVP on Huh7 sphere cells. Huh7 cells were detached and cultured in suspension, and attached and sphere cells were analyzed for the expression of csMVP by flow cytometric analysis with α-MVP. (d) Induced expression of csMVP on the surface of serum-starved A549 cells. A549 cells were serum-starved and analyzed for the expression of csMVP by flow cytometric analysis with α-MVP. (e) Graphic presentation of d. Relative expression of csMVP was measured by MFIs of flow cytometric analysis of normal (con) or serum-starved A549 cells. ***, $p < 0.005$. (f) Induced expression of csMVP on the surface of cisplatin (cispl)-treated A549 cells. A549 cells were treated with cisplatin and analyzed for the expression of csMVP by flow cytometric analysis with α-MVP. (g) Graphic presentation of f. Relative expression of csMVP was measured by MFIs of flow cytometric analysis of DMSO- or cisplatin-treated A549 cells. ***, $p < 0.005$.

Supplementary Figure 3

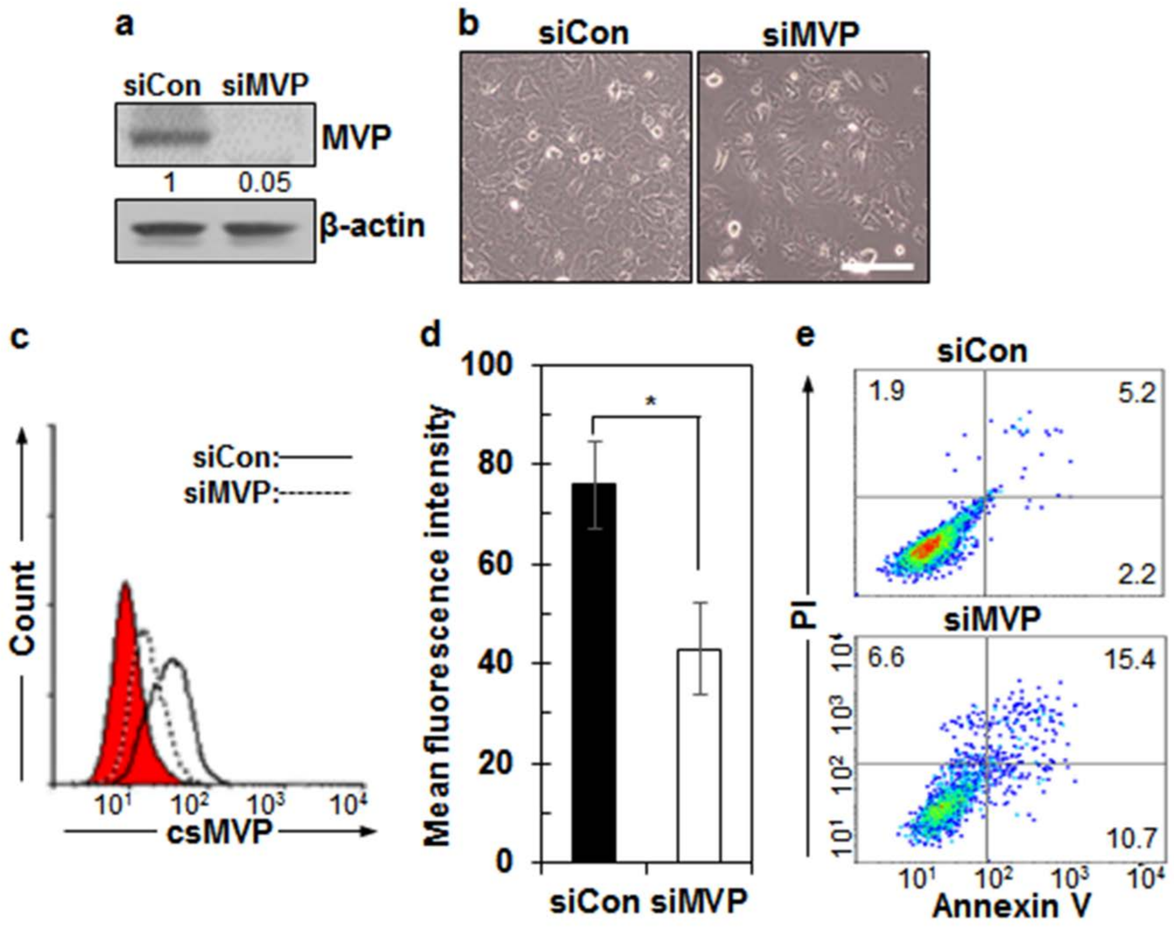

Supplementary Figure 3. MVP knockdown decreases cell surface expression of MVP and induces apoptosis of Huh7 cells. (a) Knockdown of MVP in Huh7 cells by siRNA treatment. Data are representative of three independent experiments. (b) Morphology of Huh7 cells treated control or MVP siRNAs at 48 hours after transfection. The scale bar is 50 μm. (c) Overlaid histograms showing the expression levels of csMVP in control and MVP siRNA-transfected Huh7 cells. (d) Graphic presentation of c. Relative expression of csMVP was measured (n=3) by MFIs of flow cytometric analysis of control or MVP siRNA-transfected Huh7 cells. *, p<0.05. (e) Flow cytometric analysis of the early and late apoptotic cells with annexin V and PI. Control or MVP siRNA treated Huh7 cells were stained with annexin V and PI.

Supplementary Figure 4

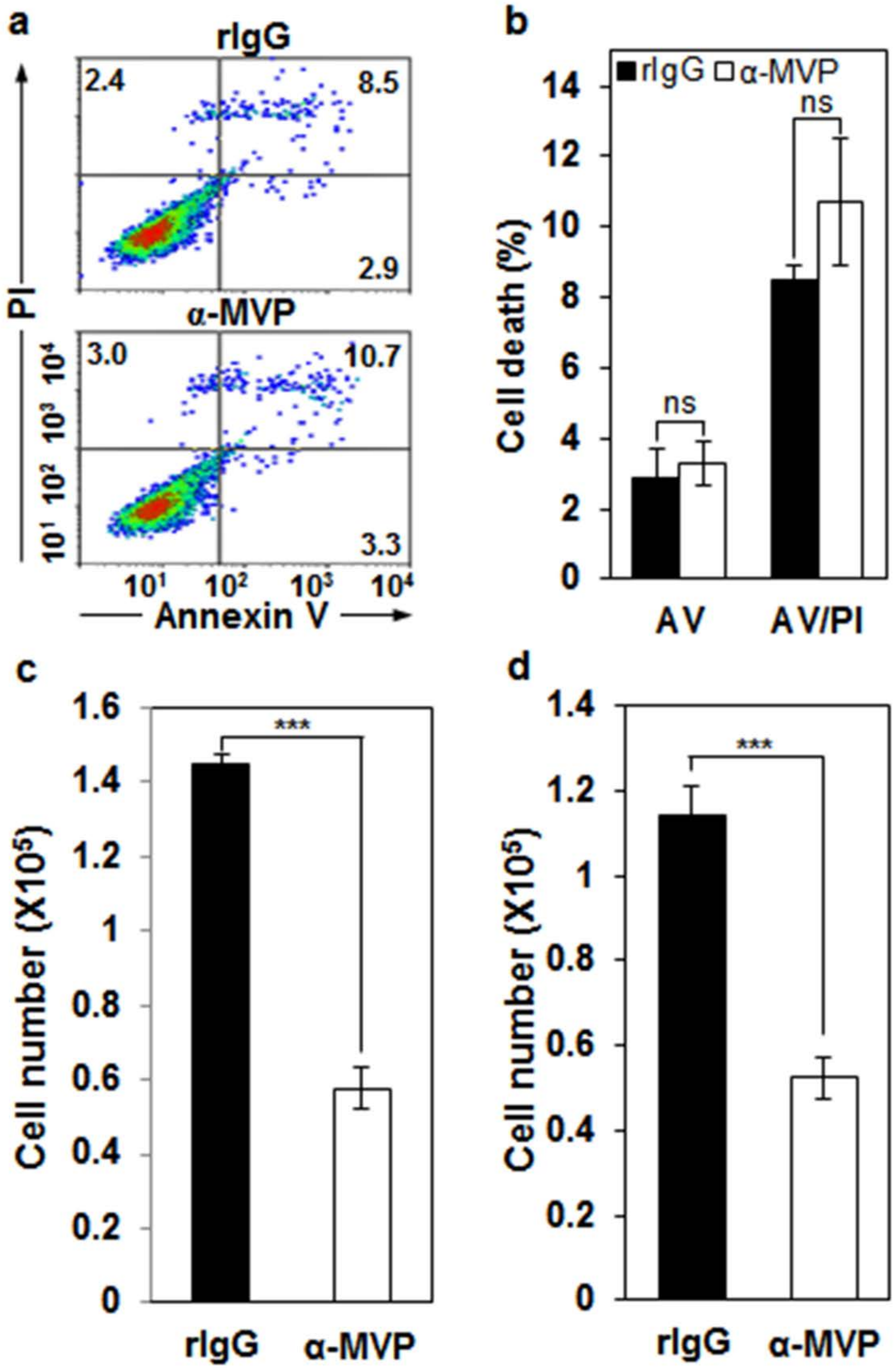

Supplementary Figure 4. Treatment with α-MVP inhibits the proliferation of HCC cells without inducing apoptosis. (a) Flow cytometric analysis of the early and late apoptotic cells with annexin V and PI. Huh7 cells treated with rIgG or α-MVP were stained with annexin V-FITC amd PI. (b) Graphic presentation of percentages of single annexin V (AV) and both annexin V- and PI-positive cells (AV/PI) in rIgG- or α-MVP-treated Huh7 cells (*n*=4; ns, not significant). (c) Measurement of cell proliferation of α-MVP-treated HCC cells. Huh7 cells were incubated with control rIgG or α-MVP for 48 hours**,** and the cell numbers were determined (*n*=3) by trypan blue exclusion assay. ***, *p* <0.005. (d) HepG2 cells were incubated with rIgG or α-MVP for 48 hours**,** and the cell numbers were determined (*n*=3) by trypan blue exclusion assay. ***, *p* <0.005.

Supplementary Figure 5

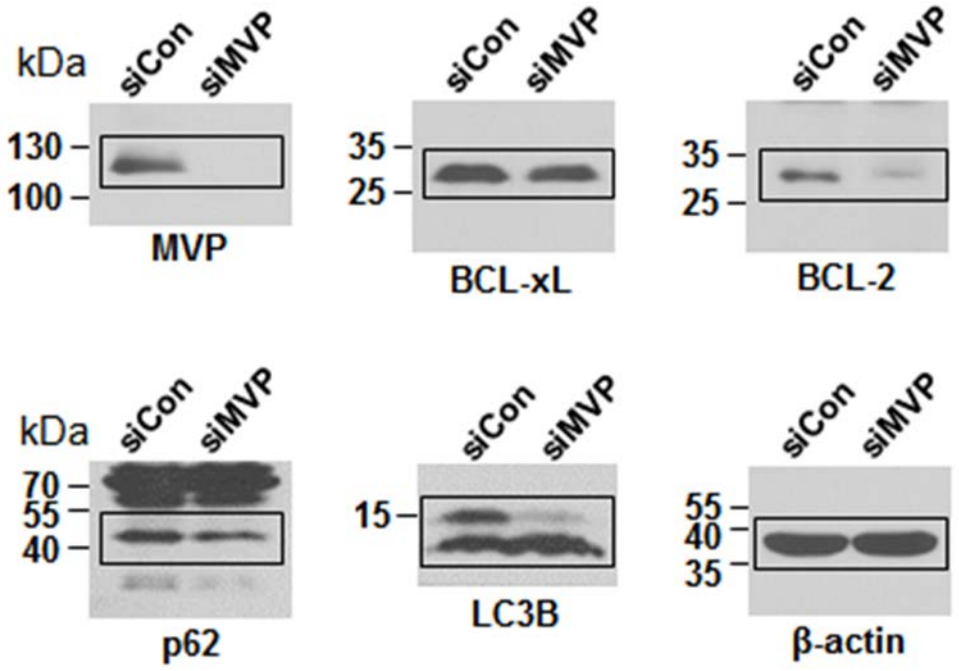

Supplementary Figure 5. Unprocessed scans of Western Blots of Fig.3c

Supplementary Figure 6

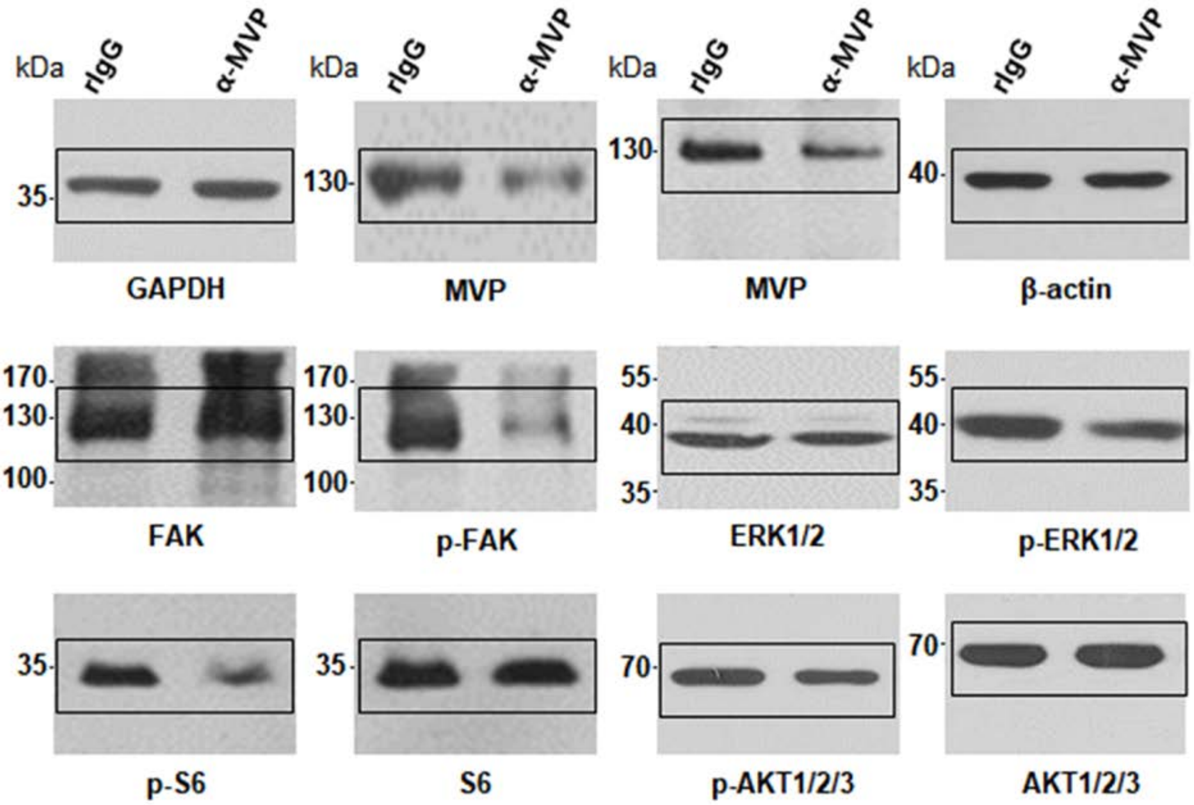

Supplementary Figure 6. Unprocessed scans of Western Blots of Fig.4

Supplementary Figure 7

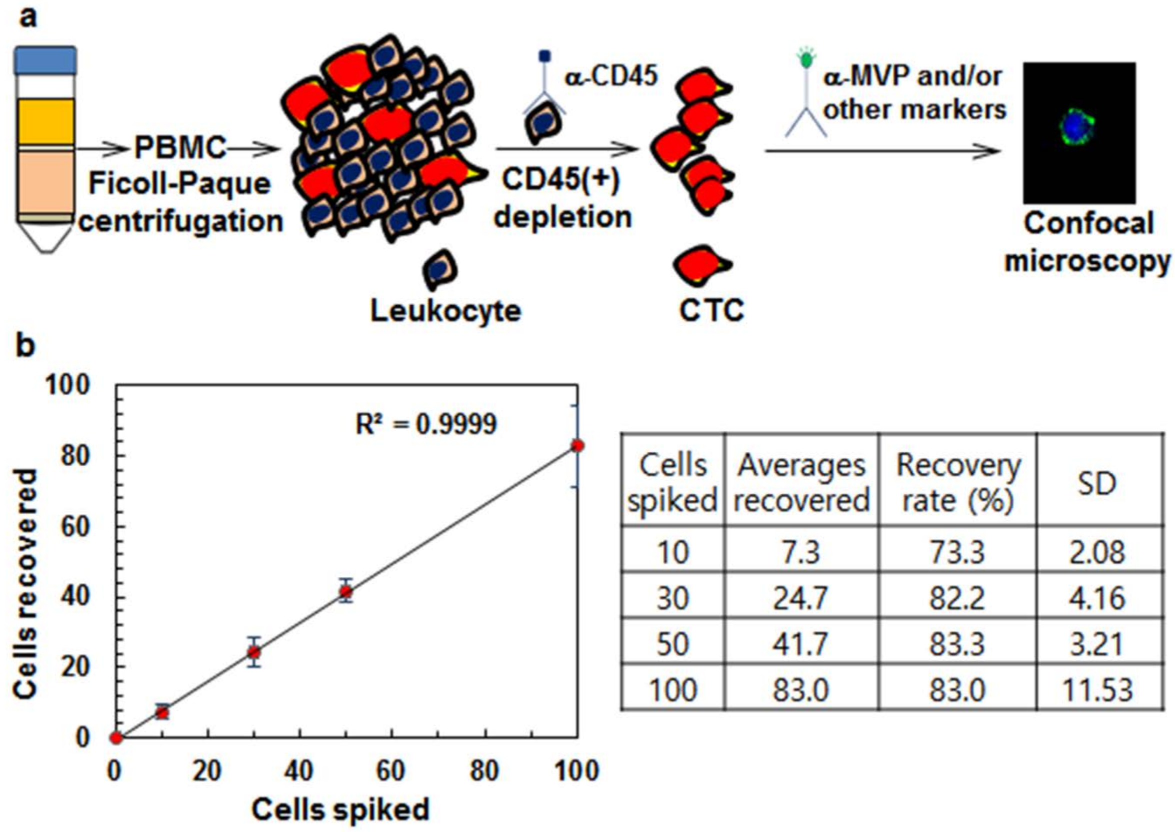

Cells spiked	Averages recovered	Recovery rate (%)	SD
10	7.3	73.3	2.08
30	24.7	82.2	4.16
50	41.7	83.3	3.21
100	83.0	83.0	11.53

Supplementary Figure 7. Spiking assay. **(**a) Schematic representation of α-MVP-mediated CTC detection. (b) Regression analysis of capture efficiency for different cell numbers of Huh7 cells spiked in human PBMCs. SD, standard deviation.

Supplementary Figure 8

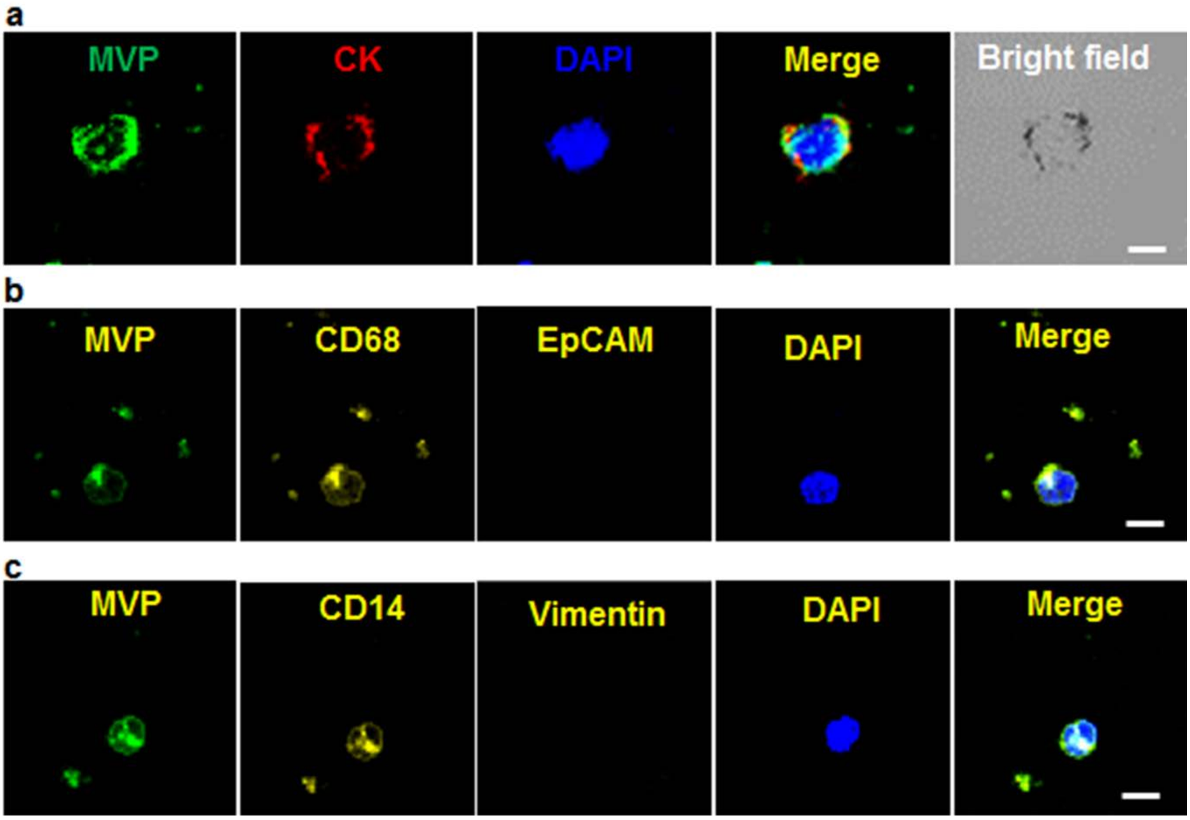

Supplementary Figure 8. Analysis of rare csMVP-positive CTCs isolated from HCC patients. (a) CD45-depleted CTCs were incubated with α-MVP (green) and anti-cytokeratin (red). (b) For triple staining for MVP, EpCAM**,** and CD68, CD45-depleted CTCs were incubated with α-MVP (green) and anti-EpCAM (red). The cells were further incubated with anti-CD68 (presented as yellow color) antibody. (c) For triple staining for MVP, vimentin**,** and CD14, cells were incubated with α-MVP (green) and anti-vimentin (red). The cells were further incubated with anti-CD14 (presented as yellow color). Nuclei were stained with DAPI (blue). The scale bar is 20 μm.

Supplementary Figure 9

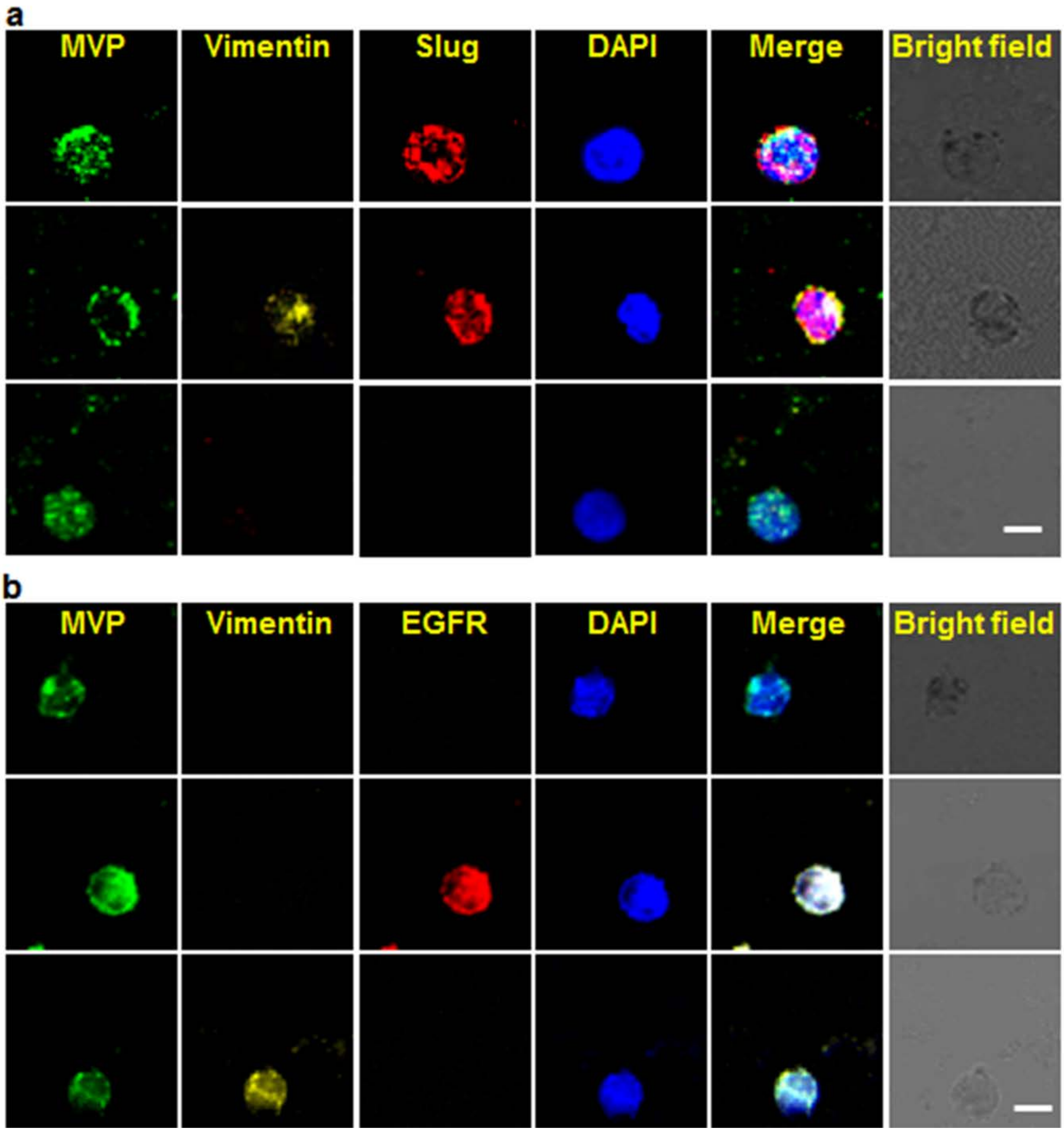

Supplementary Figure 9. csMVP-positive CTCs show EMT phenotype with Slug and EGFR expression. (a) CD45-depleted CTCs were incubated with α-MVP (green), anti-vimentin (yellow)**,** and anti-Slug (red) antibodies. Nuclei were stained with DAPI (blue). The scale bar is 20 μm. (b) CD45-depleted CTCs were incubated with anti-EGFR (red), α-MVP (green)**,** and anti-vimentin (yellow). Nuclei were stained with DAPI (blue). The scale bar is 20 μm.
